# γ-secretase directly sheds the survival receptor BCMA from plasma cells

**DOI:** 10.1038/ncomms8333

**Published:** 2015-06-11

**Authors:** Sarah A. Laurent, Franziska S. Hoffmann, Peer-Hendrik Kuhn, Qingyu Cheng, Yuanyuan Chu, Marc Schmidt-Supprian, Stefanie M. Hauck, Elisabeth Schuh, Markus Krumbholz, Heike Rübsamen, Johanna Wanngren, Mohsen Khademi, Tomas Olsson, Tobias Alexander, Falk Hiepe, Hans-Walter Pfister, Frank Weber, Dieter Jenne, Hartmut Wekerle, Reinhard Hohlfeld, Stefan F. Lichtenthaler, Edgar Meinl

**Affiliations:** 1 Institute of Clinical Neuroimmunology, Ludwig Maximilian University Munich, 81377 Munich, Germany; 2 Neuroproteomics, Klinikum rechts der Isar, and Institute of Advanced Study, Technische Universität München, 81377 Munich, Germany; 3 German Center for Neurodegenerative Diseases (DZNE), 81377 Munich, Germany; 4 Department of Rheumatology and Clinical Immunology, Charité - Universitätsmedizin Berlin and Deutsches Rheuma-Forschungszentrum Berlin-a Leibniz Institute, 10117 Berlin, Germany; 5 Department of Internal Medicine III, Klinikum Rechts der Isar, Technische Universität München, 81675 Munich, Germany; 6 Research Unit Protein Science, Helmholtz Zentrum München (GmbH), German Research Center for Environmental Health, 85764 Neuherberg, Germany; 7 Karolinska University Hospital, Division of Clinical Neuroscience, 17176 Stockholm, Sweden; 8 Department of Neurology, Klinikum Grosshadern, Ludwig Maximilian University Munich, 81377 Munich, Germany; 9 Max-Planck-Institute of Psychiatry, 80804 Munich, Germany; 10 CPC Helmholtz Zentrum München (GmbH), 81377 Munich, Germany; 11 Max-Planck-Institute of Neurobiology, 82152 Martinsried, Germany; 12 Munich Cluster for Systems Neurology (SyNergy), 81377 Munich, Germany

## Abstract

Survival of plasma cells is regulated by B-cell maturation antigen (BCMA), a membrane-bound receptor activated by its agonist ligands BAFF and APRIL. Here we report that γ-secretase directly cleaves BCMA, without prior truncation by another protease. This direct shedding is facilitated by the short length of BCMA's extracellular domain. *In vitro*, γ-secretase reduces BCMA-mediated NF-κB activation. In addition, γ-secretase releases soluble BCMA (sBCMA) that acts as a decoy neutralizing APRIL. *In vivo*, inhibition of γ-secretase enhances BCMA surface expression in plasma cells and increases their number in the bone marrow. Furthermore, in multiple sclerosis, sBCMA levels in spinal fluid are elevated and associated with intracerebral IgG production; in systemic lupus erythematosus, sBCMA levels in serum are elevated and correlate with disease activity. Together, shedding of BCMA by γ-secretase controls plasma cells in the bone marrow and yields a potential biomarker for B-cell involvement in human autoimmune diseases.

## Abstract (web summary)

B-cell maturation antigen (BCMA) regulates the survival of B cells and is essential for the maintenance of long-lived plasma cells. Here, the authors show that γ-secretase directly sheds BCMA from the cell surface and therefore regulates the number of plasma cells.

## Graphical abstract

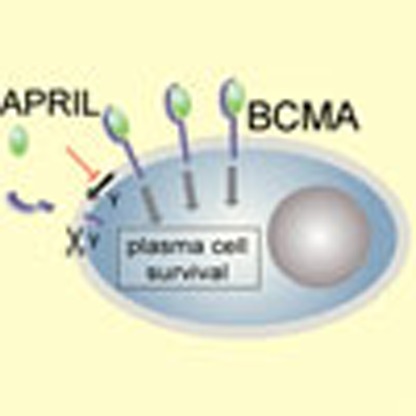

B cells are of pathogenic relevance and serve as a therapeutic target both in generalized immunopathological diseases such as systemic lupus erythematosus (SLE), and in organ-specific diseases such as multiple sclerosis (MS)^1^,^2^. Activation and survival of B cells is largely regulated via the BAFF–APRIL system that comprises three receptors (BAFF-R, TACI and BCMA (B-cell maturation antigen)) and two ligands (BAFF and APRIL)^3^. Membrane-bound BCMA (mBCMA) is expressed on some activated B cells^4^ and Ig-secreting cells^5^,^6^; it binds both BAFF and APRIL^3^. BCMA is essential for the maintenance of long-lived plasma cells^7^,^8^, an effect mediated by APRIL or BAFF^9^,^10^. These plasma cells produce IgG that protect not only against pathogens but are also critically involved in autoimmune diseases^11^,^12^. Further, mBCMA engagement on activated B cells induces MHC class II, enhancing their ability to present antigen^4^.

The BAFF–APRIL system is targeted for therapeutic intervention. In SLE, where BAFF levels in the serum are elevated, an antibody-binding BAFF is already approved^13^,^14^. On the other hand, the recombinant soluble receptor atacicept, which targets both BAFF and APRIL, unexpectedly worsened MS^15^, indicating that essential features of this system are not fully understood. Other clinical trials targeting the BAFF–APRIL system in immunopathological disorders have been launched^13^,^14^.

We aimed at identifying features of humoral immunity that might be altered in autoimmune diseases. Thereby, we found that a soluble form of BCMA (sBCMA) is regularly detectable in human blood. We then analysed sBCMA in human autoimmune diseases. In MS, sBCMA was elevated in the cerebrospinal fluid (CSF) and linked to local IgG production inside the brain. In SLE, sBCMA was systemically elevated and associated with disease activity. We went on to uncover the underlying biochemical mechanism and found that BCMA was directly shed by γ-secretase, a ubiquitous intramembranous protease. Direct shedding of a membrane protein without prior processing by another protease is a novel function of γ-secretase, which is best known for processing of amyloid precursor protein (APP) in Alzheimer's disease and Notch^16^,^17^. To analyse the functional relevance of this shedding *in vivo*, we treated mice with a γ-secretase inhibitor; this enhanced surface BCMA on plasma cells and increased their number in the bone marrow. Thus, shedding of BCMA via γ-secretase is an immunoregulatory mechanism limiting plasma cells.

## Results

### sBCMA shows local IgG production in MS and activity in SLE

**Figure 1:**
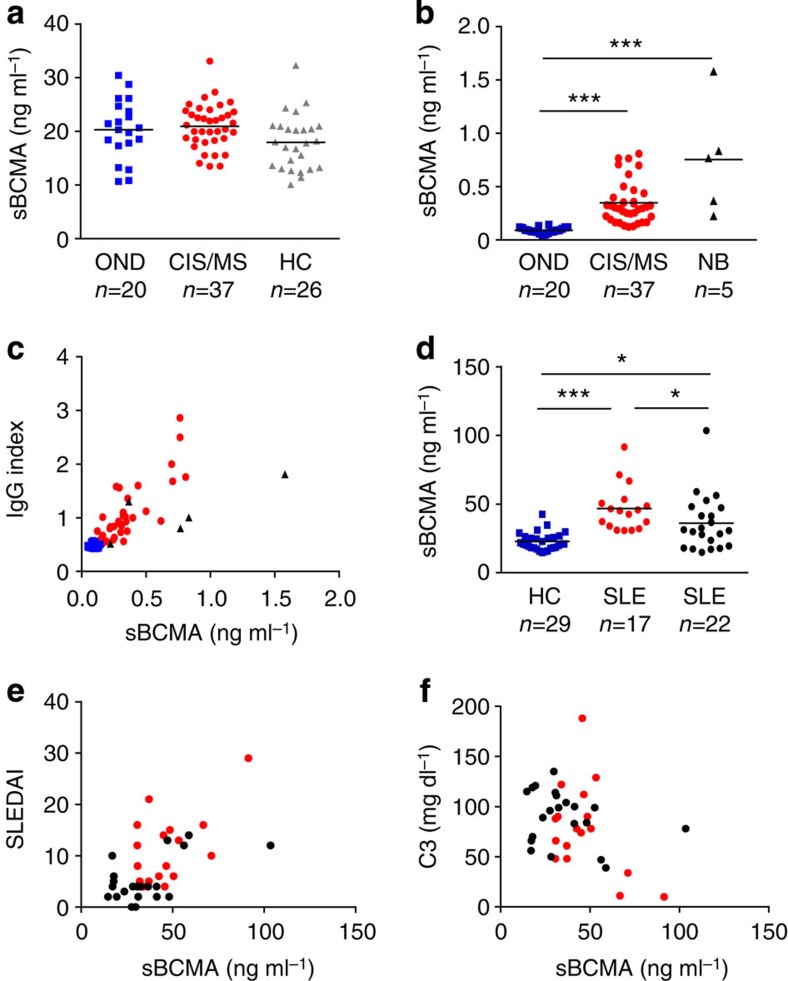
sBCMA as a biomarker. (**a**) sBCMA plasma concentrations were determined using ELISA in healthy controls (HC), patients with a clinically isolated syndrome (CIS) or MS, or other neurological diseases (OND). (**b**) sBCMA in the CSF was determined in patients with OND, CIS/MS or neuroborreliosis (NB) (***P<0.001, Kruskal–Wallis test followed by Dunn's Multiple Comparison Test). (**c**) sBCMA in the CSF correlated strongly with the intrathecal IgG production represented by the IgG Index. This correlation was evident when all analysed CSF samples were considered (*P*<0.0001, *r*=0.85) and in the MS/CIS group (*P*<0.0001, *r*=0.77, Spearman rank correlation, CIS/MS *n*=36; OND *n*=20, NB *n*=5). (**d**) sBCMA in the serum was determined with ELISA in HC, untreated (red) and treated (black) patients with SLE. sBCMA was elevated in SLE patients and in the untreated SLE patients compared with the treated patients (***P<0.001 and *P<0.05, Kruskal–Wallis test followed by Dunn's Multiple Comparison Test). (**e**) sBCMA in the serum of SLE patients correlated strongly with disease activity quantified with SLE disease activity index (SLEDAI; *P*<0.001; *r*=0.54, Spearman correlation). (**f**) sBCMA in the serum of SLE patients inversely correlated with the level of the complement factor C3 (*P*=0.0374, *r*=−0.29, Spearman correlation). Bars represent means.

We found sBCMA as a regular component of human blood from healthy subjects (Fig. 1a). In MS, sBCMA was elevated in the CSF, but not in the blood (Fig. 1a,b). sBCMA levels in the plasma and serum were very similar as seen in 14 controls (Supplementary Fig. 1a). Levels of sBCMA in the CSF correlated strongly with local IgG production in MS patients (Fig. 1c). This strong correlation was confirmed in a second cohort of 25 MS patients (*P*<0.0001, *r*=0.80, Spearman rank correlation). Also in neuroborreliosis, an infectious neuroinflammatory disease characterized by chronic IgG production within the CNS, sBCMA levels were increased and correlated with local IgG production (Fig. 1b,c). In CSF from MS patients, there was a slight inverse correlation of sBCMA with its high-affinity ligand APRIL (*r*=−0.35, *P*=0.033, Spearman rank correlation), but no association to the plasma level of sBCMA (*r*=0.07, *P*=0.667, Spearman rank correlation). We analysed effects of immunosuppressive treatment on sBCMA levels longitudinally in two cohorts of MS patients. Natalizumab, which blocks entry of lymphocytes into the CNS^18^ and local IgG production^2^, reduced sBCMA in the CSF (Supplementary Fig. 1b). High-dose steroids for treatment of acute relapses reduced serum sBCMA levels transiently (Supplementary Fig.1c).

In SLE, serum levels of sBCMA were elevated as seen in an untreated and in a treated cohort (Fig. 1d). Immunosuppressive treatment of SLE patients reduced sBCMA levels (Fig. 1d). We noted a strong correlation of serum sBCMA and disease activity (Fig. 1e) and an inverse correlation with the paraclinical marker complement factor 3 (Fig. 1f). There was a trend (*P*=0.0767, *r*=0.23, Spearman rank correlation) for correlation between anti-dsDNA titre and sBCMA in these SLE patients. Serum BAFF levels were elevated in our SLE cohort (mean=0.61±0.13 ng ml^−1^ in healthy controls; mean=2.11±4.32 ng ml^−1^ in SLE patients, *P*=0.0004, Wilcoxon–Mann–Whithney test), and correlated with sBCMA levels (*P*=0.0013, *r*=0.47, Spearman rank correlation).

### BCMA is shed during differentiation to Ig-secreting cells

**Figure 2:**
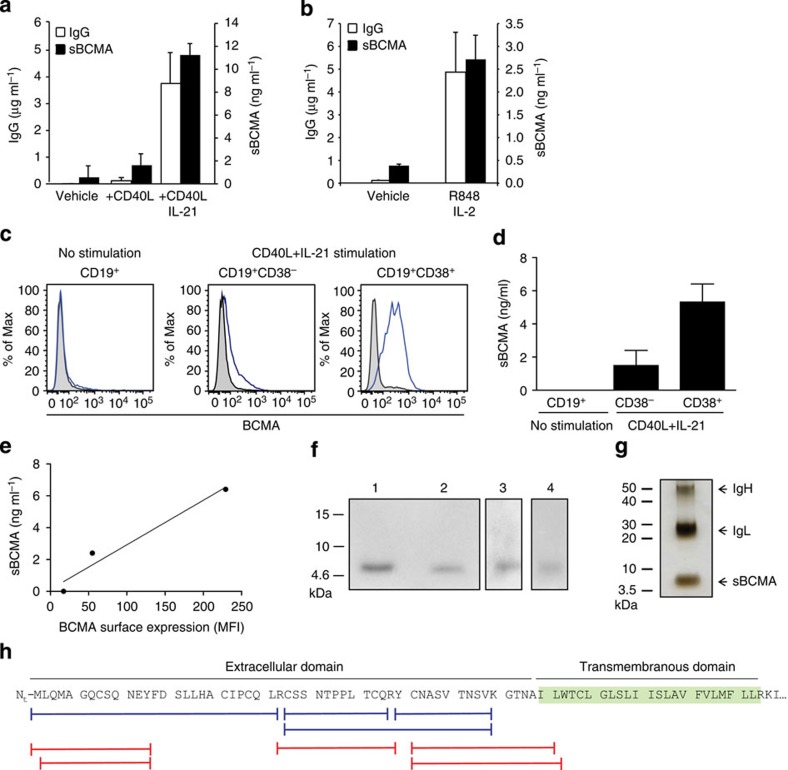
sBCMA is released when B cells differentiate towards plasma cells and comprises the extracellular domain plus part of the transmembranous region of BCMA. (**a**) Human purified B cells were activated for 5 days as indicated; IgG and sBCMA in the supernatant were quantified using ELISA. Combined data of three independent experiments (mean±s.e.m., *P*=0.0073, paired *t*-test). (**b**) PBMCs were stimulated with R848+IL-2 for 7 days. IgG and sBCMA in the supernatant were quantified using ELISA. Combined data of three independent experiments (mean±s.e.m., *P*=0.0227, paired *t*-test). (**c**–**e**) Human purified B cells were stimulated with CD40L+IL-21. (**c**) surface BCMA was measured using flow cytometry on unstimulated B cells, CD19^+^CD38^−^ cells and CD19^+^CD38^+^ cells. (**d**) Sorted CD38^+^ and CD38^−^ cells were cultured for another 24 h and the amount of shed sBCMA was measured using ELISA, combined data of two independent experiments. (**e**) Correlation between sBCMA release and surface expression of BCMA for a single replicate. (**f**,**g**) sBCMA was immunoprecipitated from supernatant of plasmacytoma cells (**f**, lanes 1, 2), serum (**f**, lane 3) and from supernatant of human purified B cells cultured with CD40L plus IL-21 (**f**, lane 4) with anti-BCMA monoclonal antibodies (mAbs) A7D12.2 (**f**, lanes 1 and 4) or C12A3.2 (**f**, lane 2) or goat-anti-BCMA (**f**, lane 3). Western blot analysis for BCMA (**f**) and silver staining of sBCMA immunoprecipitated from plasmacytoma supernatant (**g**) was performed. (**h**) The band at 6 kDa (from **g**) and sBCMA obtained using immunoprecipitation were analysed with mass spectrometry. The aa sequences of BCMA and peptides identified after tryptic (blue) or chymotryptic (red) digestion are shown. No peptide was detected with a C-terminal aa that was not a site for either tryptic or chymotryptic cleavage, indicating that the precise cleavage site of γ-secretase within the membrane needs to be identified.

Since mBCMA is known to be expressed on some activated B cells and Ig-secreting cells^5^,^6^, we analysed the release of sBCMA by primary human B cells. We applied two different protocols to activate human primary B cells in order to differentiate them towards Ig-secreting cells. First, purified blood-derived human B cells were activated via CD40L and further differentiated to Ig-secreting cells by adding interleukin (IL)-21 (ref. 5). While human B cells activated via CD40L alone released low, but detectable, levels of sBCMA, this was strongly enhanced by addition of IL-21 (Fig. 2a). In the second primary B-cell culture system we activated peripheral blood mononuclear cells (PBMC) with the TLR7+8 ligand R848 and IL-2, which induces differentiation of human memory B cells to Ig-secreting cells^19^. Again, differentiation towards IgG-secreting cells was accompanied by the appearance of sBCMA (Fig. 2b).

After activation with CD40L+IL-21, two B-cell populations, CD19^+^CD38^−^ and CD19^+^CD38^+^, cells could be distinguished. We directly compared mBCMA and release of sBCMA by these different B-cell subsets. While mBCMA was absent on unstimulated B cells, CD19^+^CD38^−^ cells weakly and CD19^+^CD38^+^ cells strongly expressed mBCMA (Fig. 2c). We sorted CD38^+^ and CD38^−^ cells, cultured them for another 24 h without further stimulation and determined the amount of shed sBCMA (Fig. 2d). This revealed a close correlation between released sBCMA and surface expression of mBCMA (Fig. 2e).

The transcription of *BCMA* in these B-cell subsets was further substantiated with qPCR. *BCMA* transcript levels in the CD38^+^ cells reached 15.9±5.2% peptidyl-prolyl isomerase A (PPIA), while *BCMA* expression in the CD38^−^ cells subset accounted for 2.5±1.3% PPIA (mean±s.e.m. of three independent replicates). Thus, in these B-cell subsets BCMA surface expression reflected *BCMA* transcription.

We also analysed BCMA shedding in tumour cell lines and transfectants. The human plasmacytoma cell line JK-6L spontaneously shed sBCMA (Supplementary Fig. 2a). In BCMA-transfected HeLa cells surface expression of mBCMA was accompanied by release of sBCMA (157±6 ng ml^−1^) without requiring any further stimulus. HeLa cells did not secrete detectable amounts of APRIL or BAFF, neither spontaneously nor after transfection with BCMA or an empty vector. Together, our observations with primary human B-cell cultures, plasmacytoma cells and BCMA-transfected cells indicate that release of sBCMA is a direct consequence of surface expression of mBCMA; it does not require additional stimulation or ligand binding.

### sBCMA comprises extracellular and intramembranous part

sBCMA was isolated by immunoprecipitation from the supernatant of primary Ig-secreting cells, plasmacytoma cells or serum; in all these sources, sBCMA had a molecular weight (MW) of ∼6 kDa as seen using western blot analysis (Fig. 2f). This size was confirmed when silver staining was applied to detect material obtained by immunoprecipitation with anti-BCMA from the supernatant of plasmacytoma cells (Fig. 2g). This corresponds to the extracellular part of BCMA (54 amino acid (aa), calculated MW 5.8 kDa). Unexpectedly, mass spectrometry revealed that sBCMA comprised not only the complete extracellular domain with an intact N terminus, but also part of the transmembrane region (Fig. 2h). This indicated that it was released by an intramembranous protease.

### γ-secretase inhibitors block BCMA shedding from B cells

**Figure 3:**
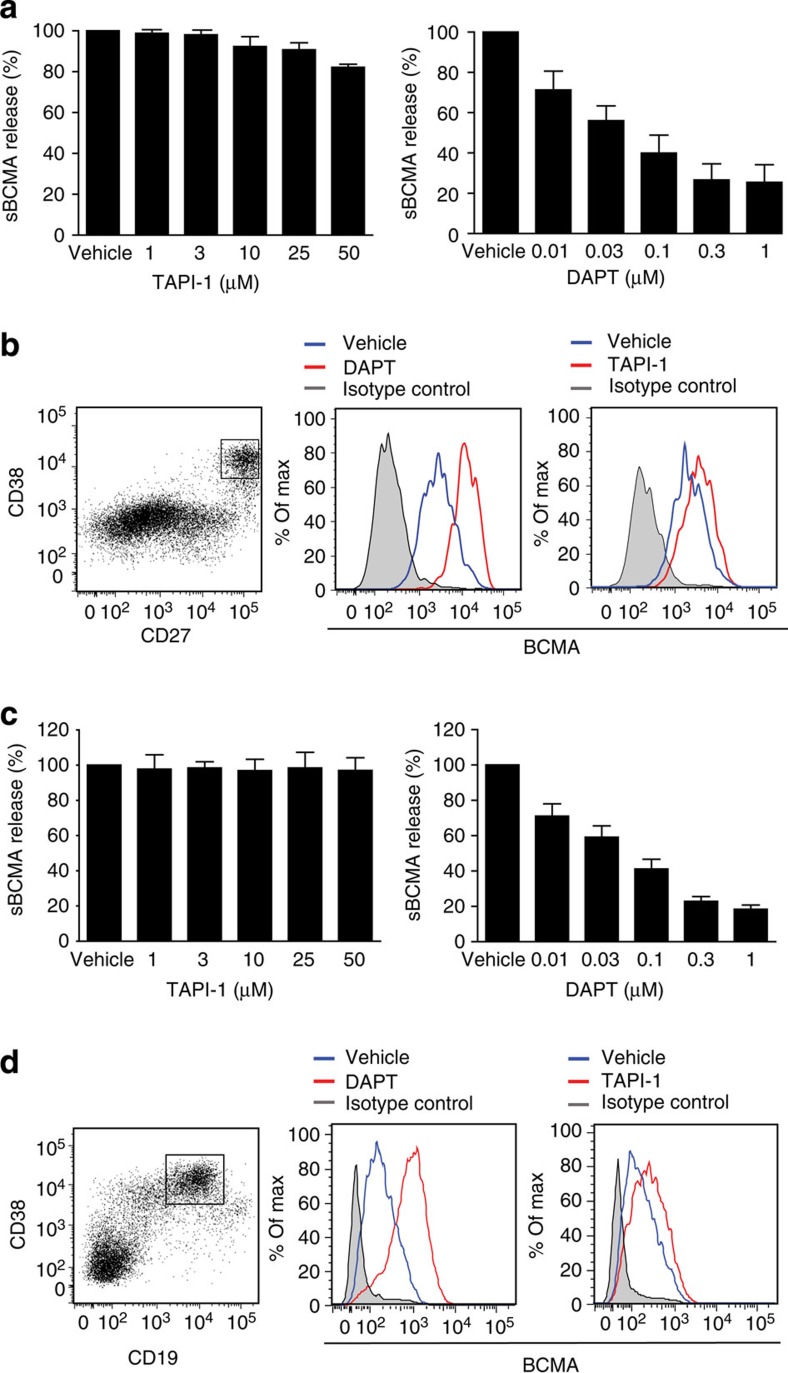
γ-secretase inhibitor DAPT reduces release of sBCMA and enhances surface expression of BCMA on activated human B cell. (**a**,**b**) Human B cells were differentiated into Ig-secreting cells via CD40L+IL-21. (**a**) Release of sBCMA on treatment with DAPT or TAPI-1 was measured using ELISA. sBCMA release was normalized to the amount of sBCMA shed under vehicle conditions, which was set as 100%. Combined data of three independent experiments (mean±s.e.m.). (**b**) These activated primary human B cells were subgrouped on the basis of expression of CD27 and CD38. A high surface expression of BCMA was seen on the CD27^++^CD38^+^ subset. Surface expression of BCMA was enhanced using DAPT treatment (1 μM), while TAPI-I (50 μM) had little effect. (**c**,**d**) Human PBMCs were stimulated with R848+IL-2 for 7 days. (**c**) Release of sBCMA on treatment with DAPT or TAPI-1 was measured using ELISA. sBCMA release was normalized to the amount of sBCMA shed under vehicle conditions, which was set as 100%. Combined data of three independent experiments (mean±s.e.m.). (**d**) High surface expression of BCMA was seen on the CD19^+^CD38^+^ subset; this was further enhanced by DAPT (1 μM), while TAPI-I (50 μM) had little effect.

Since mBCMA is a type-I oriented transmembrane protein with an extracellular N terminus, γ-secretase was a candidate for its intramembranous cleavage. We applied the γ-secretase inhibitor DAPT and compared it with the metalloprotease inhibitor TAPI-1, which reduces the shedding of other TNFR-SF members. We activated human B cells either via CD40L+IL-21 (Fig. 3a,b) or via R848+IL-2 (Fig. 3c,d), and used both fluorescence-activated cell sorting (FACS) and enzyme-linked immunosorbent assay (ELISA) as read-out systems to quantify mBCMA and sBCMA. DAPT blocked the release of sBCMA even at low concentrations, while TAPI-1 had little or no effect (Fig. 3a,c). After CD40L+IL-21 application, a high surface expression of mBCMA was noted in the CD27^++^CD38^+^ subset (Fig. 3b), previously classified as late plasmablasts^20^. DAPT enhanced surface expression of mBCMA in these cells, while TAPI-I had little or no effect (Fig. 3b). When human PBMCs were activated with R848+IL-2, ∼20% of the cells were CD19^+^CD38^+^ after 7 days (Fig. 3d). These cells strongly expressed mBCMA on their surface and this was greatly enhanced by the γ-secretase inhibitor DAPT; again, TAPI-I had little effect (Fig. 3d). Similar to primary human B cells, we observed differential effects of DAPT and TAPI-1 on the release of sBCMA and surface expression of mBCMA on human plasmacytoma cells (Supplementary Fig. 2a,b).

Further, we compared the effect of transition (LY-411575-I and LY685,458) and non-transition state (DAPT and RO4929097) inhibitors of the γ-secretase on BCMA shedding from human B cells. Human PBMCs were first stimulated with R848+IL2 for 7 days, and then CD19^+^ B cells were positively selected and cultured overnight in the absence of these γ-secretase inhibitors. We found that RO4929097, LY-411575-I and LY685,458 had similar effects as DAPT on the shedding of mBCMA as seen with both read-out systems, FACS and ELISA (Supplementary Fig. 3).

### γ-secretase directly sheds BCMA

**Figure 4:**
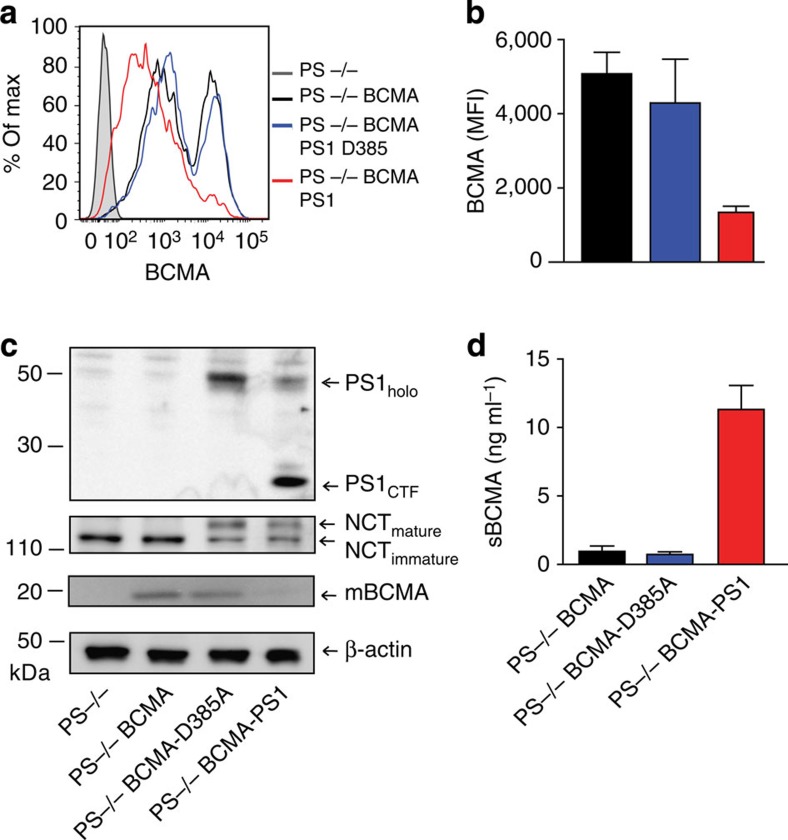
Release of sBCMA requires active presenilin. (**a**–**d**) Presenilin-deficient MEF cells (PS−/−) were transduced with human BCMA (PS−/− BCMA) and then with wild-type PS1 (PS−/− BCMA PS1) or with a catalytically inactive mutant (PS−/− BCMA PS1-D385A). BCMA surface expression (**a**,**b**) and sBCMA release (**d**) were determined. In **a**, a representative experiment is shown; in **b**,**d**, mean±s.e.m. of four independent experiments is given (respectively, *P*=0.0313 and *P*=0.0033, paired *t*-test). (**c**) Cells used in **a**,**b**,**d** were analysed by immunoblotting for expression of full-length PS1 (PS1_holo_), for autoendoproteolysis of PS1 generating a C-terminal fragment (PS1_CTF_) reflecting an active state of the γ-secretase, for maturation of nicastrin (NCT) and for full-length BCMA (mBCMA).

**Figure 5:**
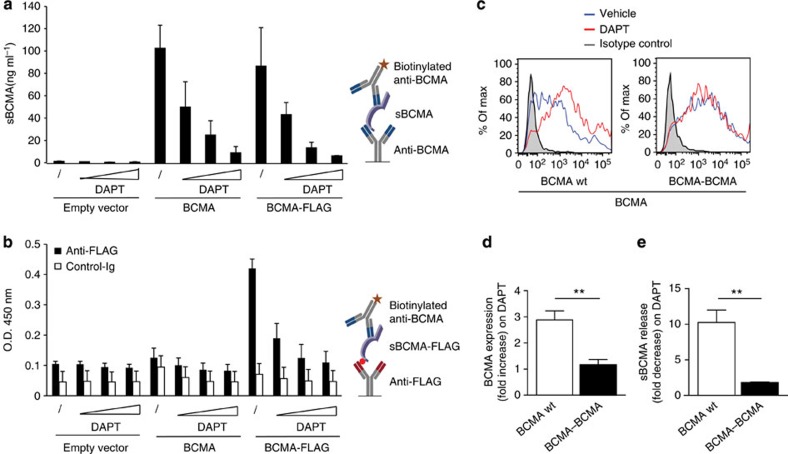
Release of sBCMA occurs without prior N-terminal trimming and is facilitated by the short extracellular domain of BCMA. (**a**,**b**) HeLa cells were transfected with plasmids coding for full-length human BCMA or BCMA with an N-terminal FLAG and then cultured with increasing amounts of the γ-secretase inhibitor DAPT (0.02, 0.1 and 0.5 μM). Twenty-four hours after transfection supernatants were harvested and the released sBCMA was analysed using ELISA. In (**a**), ELISA wells were coated with anti-BCMA, and in (**b**) with anti-FLAG or a control IgG (anti-myelin oligodendrocyte glycoprotein (MOG) 8.18 C5), both were developed with anti-BCMA. Schemes of the ELISAs are shown on the right. Combined data of two independent experiments (mean±s.e.m.). (**c**–**e**) Human BCMA wild type (wt) or BCMA–BCMA with a doubled extracellular domain of BCMA were transfected into HEK293T cells. (**c**,**d**) Surface expression of BCMA was determined in the absence or presence of the γ-secretase inhibitor DAPT (1 μM). (**d**) The combined data of three experiments (*P*=0.0049, **P<0.01, unpaired *t*-test). (**e**) The effect of DAPT on the released sBCMA after transfection with BCMA wt or BCMA–BCMA (mean±s.e.m. of three experiments), *P*=0.0081, **P<0.01, unpaired *t*-test.

Presenilin (PS)1 or PS2 is the catalytical component of the γ-secretase complex^14^,^15^. To finally prove that sBCMA is released by γ-secretase, we switched from B cells to mouse embryonic fibroblasts (MEF) deficient for both PS1 and PS2 (PS−/−)^21^. These MEF cells were transduced with full-length human BCMA plus either wt PS1, or its catalytically inactive mutant D385A (ref. 22). Cleavage of mBCMA and release of sBCMA occurred only in the presence of an active γ-secretase complex as seen with FACS, ELISA and western blot analysis (Fig. 4a–d). To date, γ-secretase is known to cleave only membrane proteins after a previous cut by other proteases^16^,^17^. This was in conflict with our mass spectrometry analysis indicating that sBCMA had an intact N terminus (Fig. 2e). Therefore, we used an additional experimental approach to assure that γ-secretase cleaved mBCMA without prior N-terminal trimming. We transfected a cDNA construct coding for BCMA tagged with an N-terminal FLAG. Using an ELISA with anti-FLAG for coating and anti-BCMA for detection, we found the release of sBCMA with N-terminal FLAG, which was blocked by the γ-secretase inhibitor DAPT (Fig. 5a,b). This provides further evidence that γ-secretase sheds BCMA without prior N-terminal trimming by another protease.

To analyse the importance of the length of the short extracellular domain of BCMA (54 aa) for its direct cleavage by γ-secretase, we doubled its extracellular part (variant BCMA–BCMA) with an extracellular part of 108 aa. We transfected wild-type BCMA and BCMA–BCMA in HEK293T cells. While the γ-secretase inhibitor DAPT strongly enhanced the surface expression of transfected wild-type mBCMA, DAPT had little or no effect on surface expression of BCMA–BCMA (Fig. 5c,d). Similarly, when we measured the released sBCMA, we found that DAPT strongly decreased its release from wild-type BCMA, but had little effect on shedding of BCMA–BCMA (Fig. 5e). Thus, the short extracellular domain of BCMA facilitates its direct cleavage by γ-secretase.

### γ-secretase regulates NF-κB-mediated activation via BCMA

**Figure 6:**
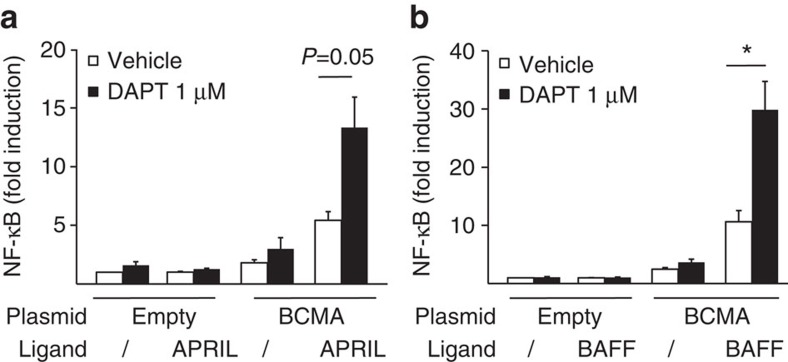
γ-secretase regulates BCMA-mediated NF-κB activation. (**a**,**b**) HEK293T cells were transfected with full-length human BCMA or an empty vector. DAPT, APRIL (**a**) or BAFF (**b**) were added, and NF-κB activation was determined. Combined data of three independent experiments (mean±s.e.m., *P<0.05, paired *t*-test).

The reduced surface expression of mBCMA mediated by γ-secretase was linked to a reduced binding of APRIL (Supplementary Fig. 2c). To analyse whether the DAPT-increased mBCMA expression was accompanied by enhanced cellular responsiveness, we analysed NF-κB activation in BCMA-transfected HEK cells. DAPT increased BCMA-mediated NF-κB activation induced via APRIL (Fig. 6a) and also via BAFF (Fig. 6b).

### sBCMA acts as a decoy inhibiting APRIL *in vitro*

**Figure 7:**
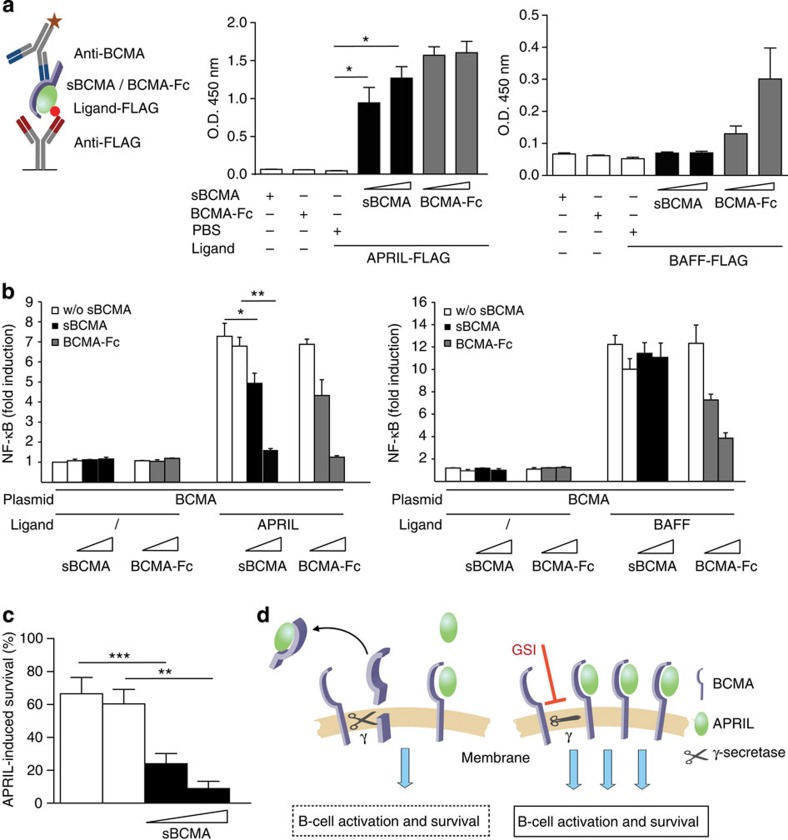
sBCMA is a decoy for APRIL *in vitro*. (**a**) A scheme of the ELISA is shown on the left; it detects BCMA–APRIL–FLAG (left panel) or BCMA–BAFF–FLAG (right panel) complexes, but neither BCMA nor APRIL–FLAG nor BAFF–FLAG alone alone (*P<0.05, paired *t*-test). sBCMA was derived from the supernatant of plasmacytoma cultured under serum-free conditions. Combined data of three independent experiments (mean±s.e.m.). (**b**) HEK293T cells were transfected with human BCMA and activated with APRIL (left panel) or BAFF (right panel). sBCMA (50 and 200 ng ml^−1^) was applied as indicated. sBCMA and control supernatant were obtained as mentioned above. BCMA-Fc (50 and 200 ng ml^−1^) was used as a positive control. Combined data of three independent experiments (mean±s.e.m., *P<0.05; **P<0.01 paired test). (**c**) Murine B cells were activated via anti-IgM and cultured for 2 days with APRIL in the presence or absence of sBCMA (200 and 400 ng ml^−1^). APRIL-induced survival was calculated as described in the Methods section. sBCMA was obtained from supernatant from HEK293T cells transfected with full-length BCMA (black bars). Control supernatant was obtained after transfection with an empty vector (white bars). sBCMA significantly inhibited APRIL-mediated survival (***P<0.001 and **P<0.01, paired *t*-test). Combined data of six independent experiments (mean±s.e.m.). (**d**) Illustration of the consequences of sBCMA shedding by γ-secretase: left: an active γ-secretase cleaves mBCMA. This reduces the number of membrane-bound BCMA molecules and releases sBCMA, which binds its ligand APRIL functioning as a decoy. Right: γ-secretase inhibitors (GSIs) result in elevated mBCMA on the surface and increased APRIL-mediated activation and survival.

We found that human sBCMA bound APRIL, but not BAFF (Fig. 7a). This is different to mBCMA, which binds both APRIL and BAFF^3^,^23^. We found that the naturally occurring sBCMA differed from recombinant BCMA-Fc, which bound APRIL and BAFF; however, binding to APRIL was stronger (Fig. 7a). Differences between sBCMA and BCMA-Fc were also seen in NF-κB reporter assays: in HEK cells transfected with BCMA, APRIL-mediated NF-κB activation was blocked by both sBCMA and BCMA-Fc, while BAFF-mediated activation via mBCMA was suppressed only by BCMA-Fc, but not by sBCMA (Fig. 7b). Since APRIL can signal via mBCMA and TACI^3^, we transfected HEK cells also with TACI. These experiments showed that sBCMA inhibited APRIL, but not BAFF-mediated signalling via TACI (Supplementary Fig. 4). Further, sBCMA limited APRIL-mediated survival of activated primary B cells (Fig. 7c). The effects of BCMA shedding by γ-secretase are summarized in a diagram (Fig. 7d).

### γ-secretase regulates plasma cells and B cells *in vivo*

**Figure 8:**
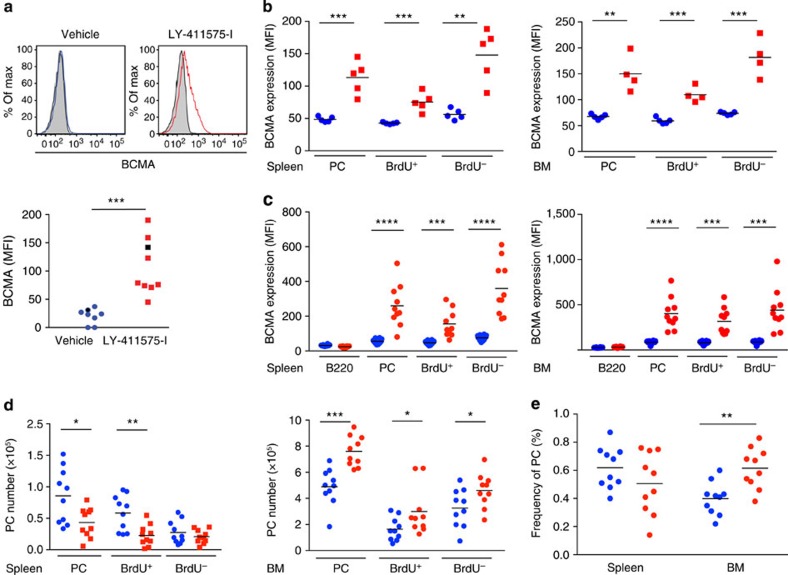
γ-secretase regulates plasma cells in mice. (**a**) Immunized (OVA–LPS in alum) C57/BL6 mice were treated with the γ-secretase inhibitor LY-411575-I or vehicle, and the surface display of BCMA in splenocytes was measured using flow cytometry 1 day later. BCMA expression on gated B220^+^CD138^+^ cells is shown, a representative example (left) and compiled data from all 17 analysed animals (mean, ***P<0.001, unpaired *t*-test; right). The black symbols on the right indicate the samples shown on the left. Closed histograms indicate isotype controls. (**b**) NZB/W mice pretreated with BrdU received the γ-secretase inhibitor LY-411575-I (red) or vehicle (blue) for 1 day. Surface expression of BCMA on all CD138^+^ plasma cells (PC) and the BrdU^+^ and BrdU^−^ PC subgroups in the spleen and bone marrow (BM) was determined using flow cytometry. (**c**–**e**) Seven-day treatment of NZB/W mice with LY-411575-I. (**c**) BCMA surface expression in the spleen and BM on B220, and BrdU^+^ and BrdU^−^ plasma cells was determined. (**d**) Absolute number of plasma cells, BrdU^+^ and BrdU^−^ plasma cells in the spleen and BM. (**e**) Frequency (% of all cells in the organ) of plasma cells in the spleen and BM. Compiled data from 10 analysed animals per group (mean; *P<0.05; **P<0.01; ***P<0.001; ****P<0.0001, unpaired *t*-test).

We tested whether cleavage of mBCMA by γ-secretase occurred also *in vivo* using two different mouse models. First, we immunized mice with ovalbumin (OVA)+lipopolysaccharide (LPS) to induce a T-dependent immune response and plasma cell differentiation. Treatment 9 days later with the γ-secretase inhibitor LY-411575-I for 1 day enhanced surface expression of mBCMA in CD138^+^ plasma cells in the spleen (Fig. 8a) and bone marrow (Supplementary Fig. 5). In the absence of a γ-secretase inhibitor, mBCMA was hardly detectable at all on the surface of murine plasma cells, in line with previous observations^8^. Second, we used an SLE model in NZB/W mice, in which germinal centres develop spontaneously^24^,^25^. In addition here, 1 day of treatment with the γ-secretase inhibitor LY-411575-I enhanced surface expression of BCMA on plasma cells in the spleen and bone marrow (Fig. 8b). To distinguish between short-lived and long-lived plasma cells, mice were fed bromodeoxyuridine (BrdU). We found that inhibition of γ-secretase for 1 day enhanced mBCMA both on BrdU^+^ plasma cells (designated also as short-lived plasmablasts) and on BrdU^−^ plasma cells (designated also as long-lived plasma cells) in the bone marrow and spleen (Fig.8b).

We then analysed the effects of extended application of the γ-secretase inhibitor and treated NZB/W mice for 7 days. This resulted again in enhanced expression of mBCMA on BrdU^+^ and BrdU^−^ plasma cells in the bone marrow and spleen (Fig. 8c). This prolonged treatment period enhanced the number of plasma cells in the bone marrow, but not in the spleen (Fig. 8d). In the spleen, the absolute number of BrdU^+^ plasma cells per organ decreased, but not their relative number (Fig. 8d,e). In the bone marrow, treatment with the γ-secretase inhibitor increased both the absolute and also the relative number of plasma cells (Fig. 8d,e).

The increase in plasma cell number in the bone marrow after inhibition of γ-secretase could be attributed to the regulation of mBCMA on plasma cells (Fig. 8, Supplementary Fig. 4) or on decoy effects of sBCMA, which we observed *in vitro* (Fig. 7b,c). To analyse possible immunoregulatory effects of the shed sBCMA *in vivo*, we produced recombinant sBCMA and applied it systemically. We prepared sBCMA as a fusion protein with α1-antitrypsin (AT; Supplementary Fig. 6a) to increase its molecular weight and therefore its half live *in vivo*. The monomeric structure of sBCMA-AT was confirmed using gel filtration (Supplementary Fig. 6b). Having shown that recombinant sBCMA-AT blocked APRIL-mediated NF-κB activation *in vitro* (Supplementary Fig. 6c), we gave sBCMA-AT three times a week for 4 weeks to NZB/W mice, which had been immunized with OVA before. The applied amount of sBCMA-AT corresponded to the dose previously used for BCMA-Fc^26^,^27^. Nevertheless, under these conditions sBCMA-AT did not modulate the total number of plasma cells, the number of OVA-specific plasma cells or the amount of circulating IgG, IgA and IgM (Supplementary Fig. 6d–f).

Notably, inhibition of γ-secretase had additional effects on B cells not expressing BCMA. Short-term treatment with the γ-secretase inhibitor reduced GC B cells (defined as CD38^int^Fas^high^) in the spleen as seen in our immunization model (Supplementary Fig. 7a,b). Moreover, after 7 days of treatment, NZB/W mice had fewer B cells in the spleen and bone marrow and showed a striking reduction of pre-B cells in their bone marrow (Supplementary Fig. 7c).

## Discussion

We report that γ-secretase directly sheds mBCMA and regulates the number of plasma cells in the bone marrow. Further, the released sBCMA reflects B-cell activation in human autoimmunity, namely compartmentalized Ig production in MS and disease activity in SLE.

When B cells are activated and differentiated towards Ig-secreting cells, they start to express mBCMA^5^,^6^, which is then shed by γ-secretase as we show here. Our experiments with activated primary human B cells, plasmacytoma cells and BCMA transfectants indicate that BCMA shedding by γ-secretase is a consequence of surface expression of mBCMA; it does not require additional activation or ligand binding; however, we cannot exclude that this shedding can be further enhanced by yet unknown mechanisms. γ-secretase is ubiquitously expressed^16^,^17^ and accessibility of its transmembranous substrates is largely regulated by the length of their extracellular part. While membrane proteins with an extracellular domain exceeding 100 aa are not efficiently cleaved, extracellular domains of ∼50 aa permit effective cleavage^28^,^29^. γ-secretase may remove protein stubs for further degradation and was hence called ‘proteasome of the membrane'^16^, although this notion is controversially discussed^29^. Probably due to these length restrictions, it has been a unifying feature of all substrates of γ-secretase (for example, NOTCH and APP) that they become accessible to cleavage only after their extracellular domain has been trimmed by another protease^16^,^17^. We now show that γ-secretase can cleave mBCMA directly and that prolonging its extracellular part greatly reduced this cleavage. Thus, constitutive cleavage of mBCMA by γ-secretase is facilitated by its short (54 aa) extracellular domain. Further we found that γ-secretase reduced BAFF and APRIL-mediated NF-κB activation via BCMA *in vitro*.

To get insight into the *in vivo* relevance of mBCMA shedding by γ-secretase, we used an immunization protocol and an SLE model with spontaneous formation of germinal centres and plasma cells^24^,^25^. We found that inhibition of γ-secretase enhances mBCMA on plasma cells in the bone marrow and spleen. This was observed in both BrdU^+^ and BrdU^−^ plasma cells indicating that both short-lived plasmablasts and long-lived plasma cells are regulated. Application of a γ-secretase inhibitor *in vivo* for 7 days enhanced the number of plasma cells in the bone marrow, but not in the spleen. We assume that the enhanced number of plasma cells in the bone marrow after γ-secretase inhibition is at least partly caused by an enhanced presence of mBCMA, since mBCMA mediates the induction of the survival protein Mcl-1 in bone marrow plasma cells^7^,^8^. The differential effect of γ-secretase inhibition on the plasma cell number in the spleen and bone marrow might be explained by the previous observation that BCMA induced high expression of Mcl-1 in bone marrow but not in spleen plasma cells^8^. In addition, BCMA−/− mice had reduced plasma cell numbers in the bone marrow, but not in the spleen^8^.

Reduction of plasma cell number in the bone marrow by γ-secretase could be based on reducing membrane-bound mBCMA or, alternatively, on the shed decoy sBCMA. We tested the decoy potential of sBCMA *in vitro* and *in vivo*. *In vitro*, sBCMA blocked APRIL, but had little or no effect on BAFF, while BCMA-Fc inhibited both BAFF and APRIL similarly. The functional features we observed for sBCMA are similar to those described previously for human recombinant monomeric BCMA, which bound and blocked only APRIL, while recombinant human dimeric BCMA-Fc bound both APRIL and BAFF^30^. Binding assays revealed an avidity contribution of dimeric versus monomeric recombinant BCMA, which resulted in *a*>1,000-fold increase in apparent affinity of BCMA binding to BAFF^30^. To get insight into the immunoregulatory capacity of sBCMA *in vivo*, we produced a recombinant variant of sBCMA (sBCMA-AT). sBCMA-AT blocked APRIL-mediated NF-κB activation *in vitro*, but did, in contrast to what was observed with BCMA-Fc previously^26^, not affect plasma cell numbers in the bone marrow when given systemically. Murine bone marrow plasma cells receive survival signals either via BAFF or APRIL^10^, which is consistent with our finding that sBCMA, which blocks mainly APRIL, has no effect on plasma cell numbers in the bone marrow. Together, we conclude that the effect of BCMA shedding on the plasma cell number in the bone marrow is largely based on the reduction of surface mBCMA. This would not exclude a function of sBCMA, especially in a mucosal environment as a decoy for APRIL, since APRIL-deficient mice had a reduced IgA response to antigens encountered via the mucosal route^31^.

We further found that inhibition of γ-secretase affected B-cell subsets *in vivo* that do not express mBCMA, such as germinal centre B cells. This might be based on a blockade of NOTCH pathways, which are known to determine T–B-cell lineage commitment^32^, maintenance of marginal zone B cells^33^, survival of GC B cells^34^ and other, not yet identified, substrates of γ-secretase, which also contribute to B-cell activation^35^. Together, our data indicate that inhibition of γ-secretase enhances the survival receptor BCMA on plasma cells, increases the number of plasma cells in the bone marrow and disturbs the B-cell compartment in the spleen.

Release of membrane receptors is a general regulatory mechanism of inflammatory responses^36^. TNFR1 is a prominent example, and the immunoregulatory features of TNFR1 shedding were previously explored *in vivo* with a knock-in mouse expressing a mutated nonsheddable TNFR1, which resulted in autoinflammatory features and enhanced experimental autoimmune encephalitis^37^. Biochemically, the release of sBCMA by γ-secretase is different from the shedding of TNFR1, which is performed by the metalloprotease ADAM17 (ref. 36), and of TACI that is shed by ADAM10 (ref. 38).

Does shedding of BCMA also occur in human autoimmune diseases? We measured sBCMA levels in a compartmentalized organ-specific autoimmune disease, MS, and in a systemic disease characterized by general activation of the B-cell compartment, SLE. A hallmark of MS is B-cell persistence inside the brain compartment with local Ig production^39^,^40^, supported by local production of BAFF by astrocytes^41^. We report that sBCMA is elevated in the CSF in MS and closely correlates with intrathecal IgG production. Thus, sBCMA in the CSF reflects the local presence of Ig-secreting cells, which are known to be present in the MS brain^2^,^39^,^40^,^42^. This view is also supported by our observation that reducing inflammation with natalizumab decreases also sBCMA in the CSF. SLE is characterized by a systemic hyperactivation of the B-cell compartment^43^ with elevated surface expression of mBCMA on circulating immune cells^44^. In this disease, we found a systemic elevation of sBCMA, which is linked to disease activity. Further studies are required to characterize sBCMA as a potential biomarker of SLE activity.

sBCMA shedding adds to the complexity of the BAFF–APRIL system. This system is highly relevant in health and disease and serves as a drug target^13^,^45^,^46^. Release and function of sBCMA are of direct relevance for clinical trials targeting BAFF, APRIL and their receptors, which are currently under way^13^. This system also affects certain haematological malignancies such as plasmacytoma^3^, where sBCMA may also serve as a biomarker^47^. Further, since γ-secretase is involved not only in Alzheimer's disease, but also in autoimmune diseases (including MS), where γ-secretase inhibitors are being tested for therapeutic benefit^48^, our findings draw attention to potential side effects of γ-secretase inhibitors related to the shedding of BCMA.

Together, our study yields three main findings. First, γ-secretase directly cleaves mBCMA. Regulation of surface display of a ligand-binding receptor is a novel function of γ-secretase. Second, γ-secretase regulates the number of plasma cells in the bone marrow. Third, the released sBCMA is a potential biomarker in human immunological diseases and could be useful for therapeutic optimization.

## Methods

### Clinical samples

In a first cohort we obtained plasma and the corresponding CSF from 37 untreated patients diagnosed with either clinical isolated syndrome (*n*=10) or MS (*n*=27) and from 20 untreated patients with other neurological diseases; further five CSF samples from neuroborreliosis patients were obtained. In a second cohort, we analysed CSF pairs from 25 additional MS patients before and about 1 year after continuous natalizumab therapy. The IgG production within the brain compartment (intrathecal) was calculated as the IgG index ((CSF IgG/CSF albumin)/(serum IgG/serum albumin)). We examined longitudinally the sera of 10 patients with MS who were treated with high doses of steroids (1,000 mg per day methylprednisolone intravenously for 3–5 days) because of a relapse. Samples were obtained directly before treatment, 3 days and 4 weeks later. We analysed serum samples from 17 untreated and 22 treated SLE patients. The treated group included patients treated with glucocorticosteroid, hydroxychloroquine, azathioprine, cyclophosphamide and mycophenolate mofetyl. Our study included 26 plasma samples and 29 serum samples of 34 healthy control donors. Detailed data of the patients included in this study can be found in Supplementary Table 1. This study was approved by the Ethics Committee of the Ludwig Maximilian University of Munich. Informed consent was obtained according to the Declaration of Helsinki.

### Cytokines and stimuli

In all stimulation and binding assays involving APRIL, we used mouse MegaAPRIL (EnzoLifeSciences, Farmingdale, NY), which binds both human and mouse BCMA and TACI; it is fused to FLAG-tag and is referred in this study as APRIL. BAFF stimulation and binding assays were performed using either recombinant human BAFF (R&D Systems, Minneapolis, MN) or recombinant human BAFF–FLAG (EnzoLifeSciences). For stimulation of PBMC and native B cells, human IL-21 (EBioscience, San Diego, CA), TLR7+8 ligand R848 (Sigma-Aldrich, St Louis, MO) and human IL-2 (R&D Systems) were used. Mouse L cells stably transfected with human CD40L were used and expression was continuously monitored.

### Antibodies and cell lines

To detect human BCMA the following antibodies were used: monoclonal antibodies A7D12.2 (IgG2b) and C12A3.2 (IgG1) (kindly provided by BiogenIdec), and the polyclonal Ab AF193 (R&D Systems). The specificity of the two monoclonal antibodies to BCMA was confirmed using flow cytometry of BCMA-transfected MEF cells (Fig. 4a) and HEK cells (Fig. 5c), immunoprecipitation (ip) and subsequent western blot (Fig. 2f,g) or mass spectrometry (Fig. 2h). Furthermore, an ELISA using A7D12.2 or C12A3.2 for coating and the polyclonal goat antibody (AF193; R&D Systems, Minneapolis, MN) for detection with recombinant BCMA (R&D Systems) as standard detected sBCMA with a sensitivity of 30 pg ml^−1^. The following monoclonal antibodies were used for surface expression analysis using flow cytometry: fluorescein isothiocyanate (FITC)-conjugated anti-CD40L (BD PharMingen, San Diego, CA), FITC-conjugated anti-CD138 (Diaclone, Besançon, France), Cy7-conjugated anti-CD27 and eFluor 450-conjugated anti-CD38 (EBioscience), Cy7-conjugated anti-CD19 (EBioscience). For triple staining of BCMA with CD27 and CD38, we used the monoclonal antibody A7D12.2 and a 647-conjugated goat-anti-mouse IgG2 Ab (Invitrogen Life Technologies, Carlsbad, CA) along with the directly labelled IgG1 antibodies to CD38 and CD27 mentioned above. For the detection of APP-derived C99, the monoclonal antibody 4G8, SIG-39220 (Covance, Emeryville, CA) was used.

Single-cell suspensions were prepared from the bone marrow (femur and tibia) and spleen. Mouse BCMA was detected using flow cytometry with biotin-conjugated anti-mouse BCMA (BAF 593; R&D Systems) along with eFluor450- or PE-Cy7-conjugated streptavidin. BrdU staining of plasma cells was performed using a BrdU Flow Kit (BD Biosciences, San Jose, CA) according to the manufacturer's protocol. The detection of plasma cells was carried out with anti-CD138-PE (clone 281-2; BD Biosciences) for surface staining and anti-kappa-Pacific Orange (clone 187.1; DRFZ) for intracellular staining. B and T cells were identified with the following anti-mouse antibodies: CD21 (clone 7E9, BioLegend, CA, USA), CD23 (clone B3B4, BioLegend), CD24 (clone M1/69, BD Biosciences), CD93 (clone AA4.1, BioLegend), CD95 (clone Jo2, BD Biosciences), CD117 (clone 2B8, BD Biosciences), IgM (clone RMM-1, BioLegend), B220 (clone RA3-6B2, DRFZ), IgD (clone 11-26c, DRFZ), GL-7 (clone GL-7, DRFZ), CD4 (clone GK1.5, DRFZ) and CD8 (clone 53-6.7, BioLegend). Identification of B-cell subsets in the spleen: B1: IgM^high^CD21^low/−^CD23^−^CD93^−^. Follicular B cells: IgM^+^CD21^+^CD23^+^CD93^−^. Marginal zone B cells: IgM^high^CD21^+^CD23^−^CD93^−^. GL-7^+^: GL-7^+^IgD^−^. Identification of B-cell subsets in the bone marrow: pro-B cells: B220^+^CD93^+^CD117^+^. pre-B cells: B220^+^CD24^+^IgM^−^IgD^−^. Immature B cells: B220^+^CD24^+^IgM^+^IgD^−^. Mature B cells: B220^+^CD24^low/−^IgM^+^IgD^+^. Cytometric analysis was performed using a FACSCanto II cytometer (BD Biosciences) and data were analysed with the FlowJo software (Tree Star Inc.). Source and working concentration of antibodies used are listed in Supplementary Table 2.

MEFs deficient for presenilin 1 (PS1) and presenilin 2 (PS2) (PS−/−) were kindly provided by Dr Bart De Strooper (Leuven, Belgium)^21^; PS−/− status was monitored using western blot analysis with the antibody PSEN1 (Epitomics, Burlingame, CA). The plasmacytoma cell line JK-6L (ref. 49) was kindly provided by Dr Silke Meister (Erlangen, Germany); expression of the surface markers CD138 and BCMA was controlled. This cell line was cultured in RPMI supplemented with 10% FCS and in serum-free conditions (Hybridoma 6 direkt from Bio&SELL, Nürnberg, Germany). Further, HeLa and HEK293T were applied in transfection experiments.

### Detection of BCMA and its ligands APRIL and BAFF

Surface expression of human BCMA was determined using flow cytometry on FACS Verse (BD Biosciences) using C12A3.2 or A7D12.2 and appropriate secondary antibodies. To measure human sBCMA in the plasma, CSF or cell culture supernatants, a sandwich ELISA with polyclonal goat antibodies was used (BCMA/TNFRSF17 ELISA Duoset; R&D Systems). To detect sBCMA with an N-terminal FLAG, the monoclonal antibody anti-FLAG M2 antibody (Sigma-Aldrich) was used for coating and the biotinylated polyclonal goat-anti-BCMA antibody (R&D Systems) for detection. As a control the anti-myelin oligodendrocyte glycoprotein monoclonal antibody 8.18C5 was used for coating. To measure APRIL in CSF, a sandwich ELISA was used (Bender MedSystem, Vienna, Austria). BAFF levels in the serum were measured using the Quantokine human BAFF Elisa kit (R&D Systems).

To assess binding of sBCMA or BCMA-Fc (kindly provided by Biogen Idec) to APRIL and BAFF, an ELISA was used with the anti-FLAG M2 monoclonal antibody (Sigma-Aldrich) for coating to bind FLAG-tagged APRIL or BAFF–FLAG. Then, sBCMA from supernatant of plasmacytoma cells, which was concentrated with Amicon Ultra 3 K devices (Merck Millipore Ltd., Ireland), or BCMA-Fc was added and incubated for 2 h at room temperature. BCMA was detected as described above.

### Immunoprecipitation and western blot analysis

Immunoprecipitation of BCMA was performed with the monoclonal antibody A7D12.2, the monoclonal antibody C12A3.2 or a polyclonal goat antibody (AF193; R&D Systems). These antibodies were coupled to Dynabeads Protein G (Life Technologies, AS, Oslo) and cross-linked with bis-sulfosuccinimidyl-suberate^3^ (Pierce Chemical Co., Rockford, IL). After successive incubation with either supernatant of plasmacytoma cells or serum, we eluted with glycine or SDS loading buffer (NuPAGE LDS Sample Buffer, Life Technologies). Cells were lysed at 4 °C for 1 h in NP-40 lysis buffer (150 mM NaCl, 50 mM Tris pH 7.5, 1% Nonidet P-40) containing complete protease inhibitor cocktail (Roche Applied Science, Penzberg, Germany). BCMA was detected by western blot analysis with the monoclonal antibody C12A3.2. Blots were developed using the mouse true blot goat anti-mouse IgG-HRP system (EBioscience) and enhanced chemiluminescence (ECL). Expression and endoproteolysis of PS1 was analysed by immunoblotting of cell lysates with the antibody PSEN1 (Epitomics); maturation of nicastrin (NCT) was evaluated by immunoblotting with the antibody N1660 (Sigma-Aldrich).

### Mass spectrometry and sample preparation

sBCMA was purified by immunoprecipitation and obtained by acidic elution. The eluate was then desalted and concentrated using StageTips, C18, microcolumns (Thermo Scientific, Bremen, Germany). Then two approaches were followed. (A) The material was digested in solution by trypsin or chymotrypsin and analysed using mass spectrometry (LTQ Orbitrap XL) as described before^50^. (B) The immunoprecipitated material was separated via an SDS gel, silver-stained and the band corresponding to BCMA as detected using western blot analysis was excised and analysed using mass spectrometry (LTQ Orbitrap XL).

### Cell culture and gene transfer

Two experimental systems were used to differentiate primary human B cells to Ig-secreting cells. First, B cells were positively selected from PBMC with CD19 MACS beads (Miltenyi Biotec, Bergisch Gladbach, Germany) and differentiated into IgG-secreting cells by coculture with CD40L-transfected mouse L cells and recombinant human IL-21 (50 ng ml^−1^). Further separation of CD19^+^CD38^+^ and CD19^+^CD38^−^ cells was performed using CD38 MACS beads. Second, PBMC were stimulated with the TLR7+8 ligand R848 (2.5 μg ml^−1^) and IL-2 (1,000 IU ml^−1^) as described^19^. Secreted IgG was quantified by ELISA (Mabtech, Nacks Strand, Sweden). To analyse the effects of DAPT and TAPI-1, these Ig-secreting cells were washed after 7 days and cultured for another 16 h with either inhibitor.

To measure APRIL-induced survival, mouse B cells (magnetically isolated from spleen using the EasySep Mouse B Cell Isolation Kit (Stemcell Technologie, Vancouver, Canada)) were cultured in 96-well microtiter plates, which were precoated with anti-IgM (5 μg ml^−1^). APRIL (100 ng ml^−1^) was added for 48 h and cross-linked with anti-FLAG monoclonal antibody (Sigma-Aldrich). Supernatants generated by HEK293T cells that had been transfected with full-length BCMA (OriGene Technologies, Inc., Rockville) or an empty control vector and therefore either contained sBCMA or did not, were added at a final sBCMA concentration of 200 ng ml^−1^ and 400 ng ml^−1^. Cell survival was quantified by flow cytometry using TO-PRO®-3 Iodide viability dye (Invitrogen Life Technologies) and APRIL-induced survival was calculated as followed: 100 x [(cell survival in presence of APRIL—cell survival without APRIL)/cell survival without APRIL].

To obtain BCMA with an N-terminal FLAG-tag, full-length human BCMA (h184) from a human BCMA cDNA clone (OriGene Technologies) was first amplified by PCR. The PCR fragment was then digested and ligated into the pCMV6-AN-DDK vector (OriGene Technologies). This construct does not contain a signal peptide, since the native BCMA also does not contain one^23^. To obtain a BCMA variant with a prolonged extracellular part, the extracellular part of human BCMA was amplified by PCR, digested and ligated at the N-terminal end of full-length human BCMA clone (OriGene Technologies). We thereby doubled the extracellular part, and called the new construct BCMA–BCMA. The plasmid peak12-SP-C99 expresses the C-terminal 99 amino acids of APP together with the APP signal peptide. C99 is a direct substrate for γ-secretase^51^. Peak12-SP-C99 was obtained by cloning SP-C99 from the pCEP4 vector into the peak12 plasmid^51^. HEK or HeLa cells were transfected with 200 ng of respective expression plasmids using lipofectamine 2,000 (Invitrogen Life Technologies).

Using lentiviral gene transduction, MEF deficient for PS1 and PS2 (PS−/−) were stably transduced with full-length human BCMA and then stably transduced with either wt PS1 or a catalytically inactive D385A mutant of PS1 (22).

### Enzyme inhibitors

We used the following γ-secretase inhibitors: DAPT (Calbiochem Merck, Darmstadt, Germany), L685,458 (R&D Systems), RO49290 (Selleckchem, Houston, TX, USA) and the steroisomer SSR of LY-411575, referred as LY-411575-I (Sigma-Aldrich). This steroisomer of the γ-secretase inhibitor LY-411575 is also a γ-secretase inhibitor as observed with the APP fragment C99 and BCMA as substrates (Supplementary Figures 3 and 8). Metalloproteases were inhibited with TAPI-1 (EMD Chemicals/Calbiochem, Inc. Gibbstown US). Corresponding concentrations of DMSO (Sigma-Aldrich) were used as vehicle controls. Reduced BCMA shedding was observed at a vehicle concentration of 0.5% DMSO, which corresponds to 50 μM TAPI-1.

### NF-κB reporter assay

To measure NF-κB activation, HEK293T cells were transiently transfected with a plasmid containing a firefly luciferase reporter gene under the control of an NF-κB transcriptional response element, a plasmid with a Renilla reniformis luciferase reporter gene for normalization and the indicated amounts of expression or control plasmids using Lipofectamine 2000 (Invitrogen Life Technologies). We used human BCMA or human TACI for transfection. To determine the effect of γ-secretase inhibition on NF-κB activation, cells transfected with BCMA were treated with DAPT or a solvent control and 6 h later stimulated with APRIL or BAFF. To analyse a possible decoy function, DAPT along with BAFF or APRIL were added to BCMA-Fc or supernatants generated by HEK293T cells that had been transfected with full-length BCMA or an empty control vector as described above. These supernatants were incubated at 37 °C for 30 min and then added to BCMA or TACI-transfected and DAPT-treated cells used for the reporter assay. 16 h after stimulation cells were harvested and cell lysates were prepared using passive lysis buffer (Promega, Madison, WI, USA) and the reporter gene activity was measured using firefly luciferase substrate (Biozym, Hameln, Germany) and renilla luciferase substrate (Promega) respectively.

### Flow cytometry sorting and quantitative RT-PCR

Human B cells were positively selected from PBMC with CD19 MACS beads (Miltenyi Biotec) and differentiated into IgG-secreting cells by coculture with CD40L-transfected mouse L cells and recombinant human IL-21 as described above. CD27^++^CD38^+^ and CD38^−^ cells were sorted using the BD FACS Aria cell sorting system (Becton Dickinson, Heidelberg, Germany). For quantitative PCR analysis, RNA was isolated using the RNeasy Micro Kit (Qiagen, Hilden, Germany) and cDNA was generated using the High Capacity cDNA Reverse Transcription Kit (Applied Biosystems, Darmstadt, Germany). To detect BCMA transcripts, primer and probes were used as published^41^. As housekeeping gene Cyclophilin A (PPIA) was used and detected with the primer and probes 43263416E from Applied Biosystems. Reactions were carried out in duplicate using TaqMan assays in combination with the TaqMan PCR Core Reagent Kit (Applied Biosystems). Samples were run in MicroAmp Optical 96-well reaction plates in a 7900HT Fast Real-Time PCR System (Applied Biosystems). Data were analysed using the SDSv2.3 software (Applied Biosystems).

### sBCMA-AT fusion protein

The cDNA of the extracellular part of mouse BCMA (sBCMA) was synthesized using the Integrated DNA Technology with a BspEI and an Age I site at the 5′ and 3′ ends, respectively, and fused to the 3′ end of human AT in the expression vector pTT5 (ref. 52). The resulting fusion between AT and sBCMA (extracellular residues from position 2 to 49 lacking the first methionine) is separated by a short flexible peptide linker TGSGSGA and terminates with hexahistidine tag to facilitate purification. The free cysteine residue on the surface of wild-type AT was replaced by a serine residue to prevent dimerization in oxidizing environments. The fusion protein was expressed in HEK293-EBNA cells as described previously^52^. MW was determined with standard Coomassie gel and the size was analysed using gel filtration with the Superose 12 HR 10/30 gel filtration column (Amersham/GE Healthcare Life Sciences, Freiburg, Germany) on a fast protein liquid chromatography column (FPLC). Function of sBCMA-AT was tested using NF-κB reporter assays.

### γ-secretase inhibitor and recombinant sBCMA-AT in mice

Two different mouse models were applied. First, 2-month-old C57/BL6 mice were immunized intraperitoneally with 100 μg OVA and 10 μg LPS in alum (a protocol based on ref. 53) and killed 10 days later. Immunized mice received an intraperitoneal dose (10 mg kg^−1^) of LY-411575-I on day 9, followed by another dose 6 h before being killed. Second, female 16- to 18-week NZB/W F1 mice were bred at the animal facility of the German Rheumatism Research Center Berlin (DRFZ) under defined, pathogen-free conditions. To distinguish between short-lived and long-lived plasma cells, we fed the mice BrdU (Sigma-Aldrich, 1 mg ml^−1^) with 1% glucose in drinking water for 2 weeks. One week after the start of BrdU feeding, mice were treated daily with the γ-secretase inhibitor LY-411575-I intraperitoneally at a dose of 10 mg kg^−1^ for 7 days. Finally, 2-month-old NZB/W mice were immunized intraperitoneally with OVA (100 μg per mouse and alum (Thermo) as adjuvant) as described^54^ and boosted at 3 months. At 4 months, mice were treated for four consecutive weeks intraperitoneally with recombinant sBCMA-AT fusion protein (200 μg three times a week). The concentration of this fusion protein in the serum of the injected mice was determined with an AT ELISA, which is specific for human AT (ab189579 from Abcam, Cambridge, UK). Single-cell suspensions were prepared from the spleen and bone marrow (femur and tibia) as described before. Cells were first incubated with anti-CD138-PE and then fixed with Cytofix/Cytoperm buffer (BD Bioscience) for 30 min. Intracellular Ig kappa light chain was detected by anti-kappa-Pacific Orange and OVA-specific antibodies were detected by intracellular OVA conjugated with FITC.

## Additional information

**How to cite this article:** Laurent, S. A. *et al*. γ-secretase directly sheds the survival receptor BCMA from plasma cells. *Nat. Commun.* 6:7333 doi: 10.1038/ncomms8333 (2015).

## Supplementary Material

Supplementary Information Supplementary Figures 1-9 and Supplementary Tables 1-2
