# Supplementary material for: γ-secretase directly sheds the survival receptor BCMA from plasma cells
Source: Nat Commun. 2015 Jun 11;6:7333. doi: 10.1038/ncomms8333 (PMC4490565; doi:10.1038/ncomms8333)
Supplement: Supplementary Information — Supplementary Figures 1-9 and Supplementary Tables 1-2 [file ncomms8333-s1.pdf]

Supplementary Figure 1

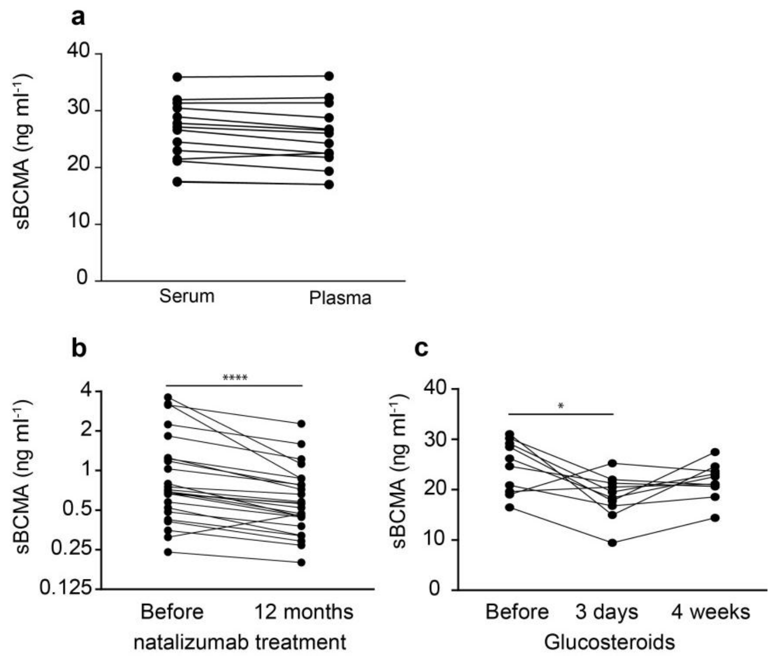

Supplementary Figure 1. Effects of immunosuppressive treatment on sBCMA levels. (**a**) sBCMA levels were measured by ELISA in plasma/serum pairs of 14 healthy controls (**b**) sBCMA in the CSF of 25 MS patients was determined before and 12 months after natalizumab treatment, which reduced sBCMA levels (****, $p<0.0001$, Wilcoxon matched-pairs ranked-test). (**c**) sBCMA levels in the serum of 10 MS patients were determined before, 3 days and 4 weeks after the beginning of high dose glucocorticoid treatment, which reduced sBCMA levels within 3 days (*, $p<0.05$, Friedman test followed by Dunn's Multiple Comparison Test).

Supplementary Figure 2

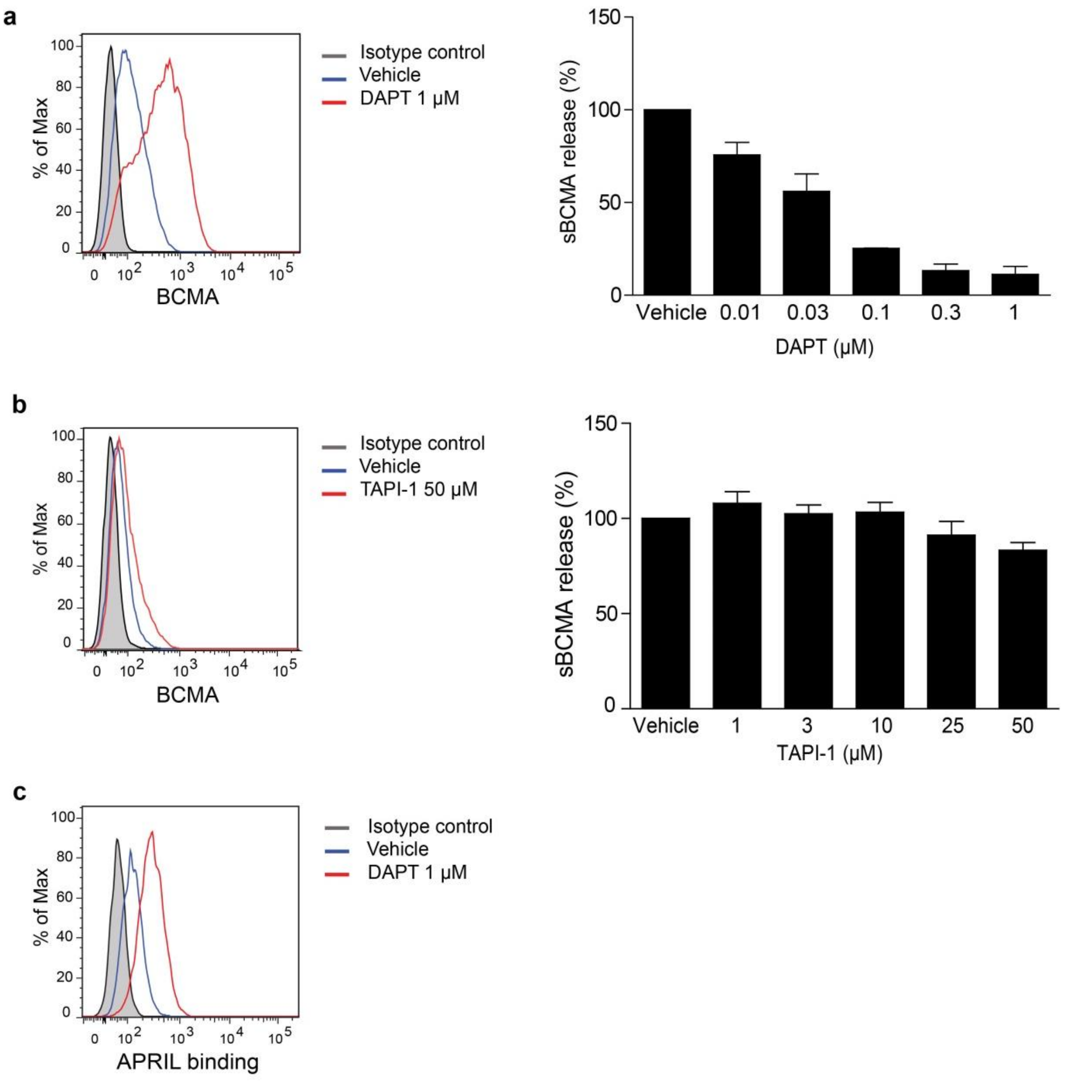

Supplementary Figure 2. γ-secretase inhibitor DAPT blocks BCMA shedding in plasmacytoma cells resulting in enhanced APRIL-binding. (**a**,**b**) The human plasmacytoma cell line JK-6L was incubated overnight with DAPT or TAPI-1. Surface expression of BCMA was measured by flow cytometry (left panels) and sBCMA in the supernatant was determined by ELISA (right panels). Combined data of 3 independent experiments (mean±s.e.m.) (**c**) BCMA ligand APRIL (FLAG-tagged) was added to plasmacytoma cells after pretreatment with DAPT or vehicle and its binding was analyzed with anti-FLAG mAb. Representative of 3 independent experiments.

Supplementary Figure 3

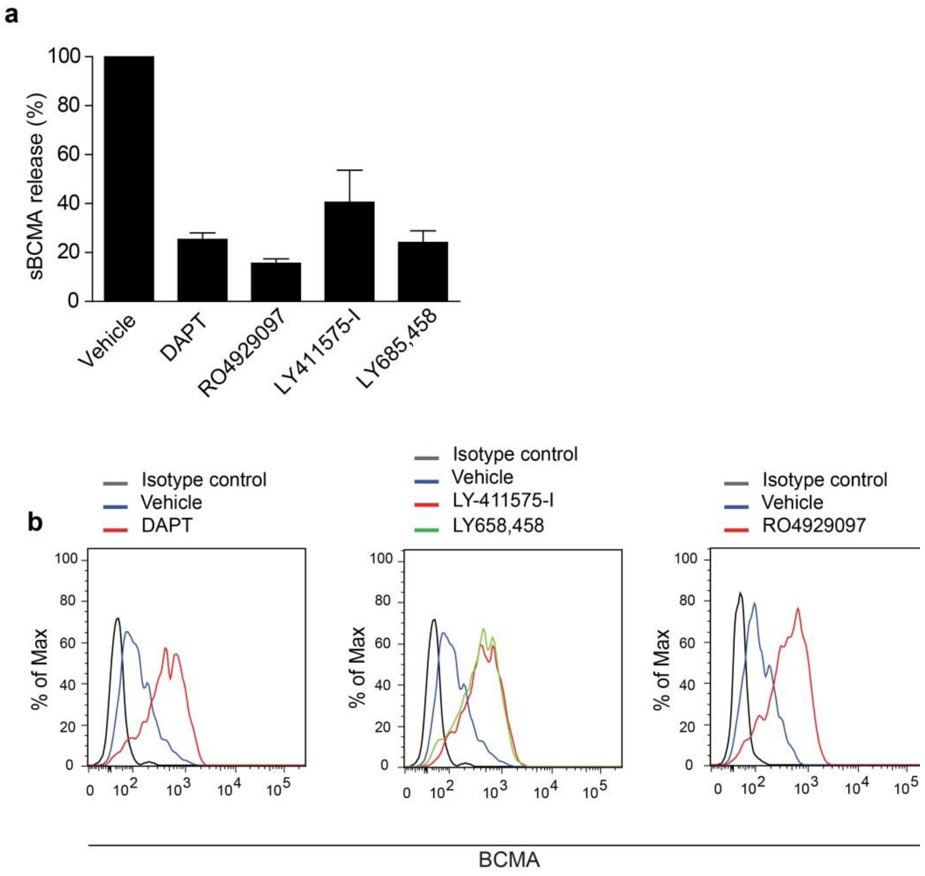

Supplementary Figure 3. Different types of γ-secretase inhibitors block the release of sBCMA. Human PBMCs were stimulated with R848 and IL-2 for 7 days and then CD19$^+$ B cells were positively selected. (**a**) After an overnight culture with the indicated γ-secretase inhibitors the released sBCMA in the supernatant was measured by ELISA, (mean±s.e.m. of three experiments) (**b**) After an overnight culture with the indicated γ-secretase inhibitors, BCMA surface expression was determined after gating on CD19$^+$CD38$^+$ Ig-secreting cells, which represented in this experiment about 50% of the CD19$^+$ cell population. Representative of 3 independent experiments. The concentrations of the γ-secretase inhibitors were DAPT 1 µM, RO4929097 10 µM, LY-411575-I 100 nM and LY685,458 1 µM. Representative of 3 independent experiments, mean±s.e.m..

Supplementary Figure 4

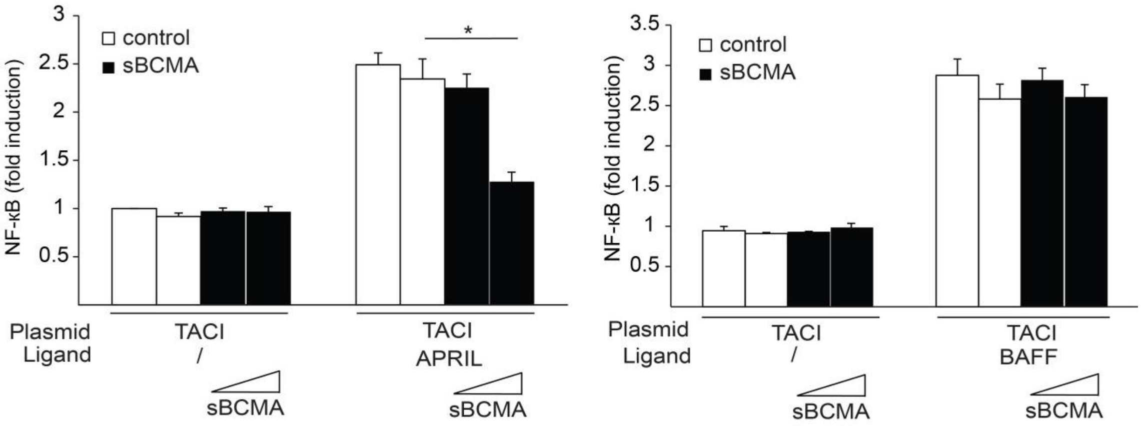

Supplementary Figure 4. sBCMA inhibits APRIL-mediated signaling via TACI. HEK293T cells were transfected with human TACI and activated with APRIL or BAFF as indicated. sBCMA (50 and 200 ng ml^{-1}) was applied as indicated. sBCMA and control supernatant were obtained from HEK293T cells that had been transfected with full-length human BCMA or an empty control vector. BCMA-Fc (50 and 200 ng ml^{-1}) was used as a positive control. Combined data of 3 independent experiments (mean±s.e.m., *, $p<0.05$, paired t-test).

Supplementary Figure 5

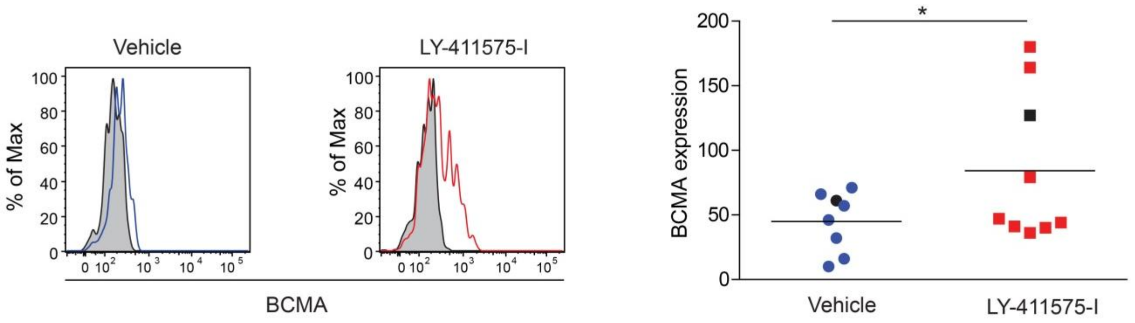

Supplementary Figure 5. Effects of γ-secretase inhibitor LY-411575-I on mBCMA on plasma cells in the bone-marrow. Immunized (OVA-LPS in alum) C57/BL6 mice were treated with the γ-secretase inhibitor LY-411575-I or vehicle, bone marrow was harvested and the surface display of BCMA was measured by flow cytometry. BCMA expression on gated $B220^{+}CD138^{+}$ cells is shown, representative example (left) and compiled data from all 17 analyzed animals (right) (mean, p=0.046 unpaired t-test). The black symbols on the right indicate the samples shown on the left. Closed histograms indicate isotype controls.

Supplementary Figure 6

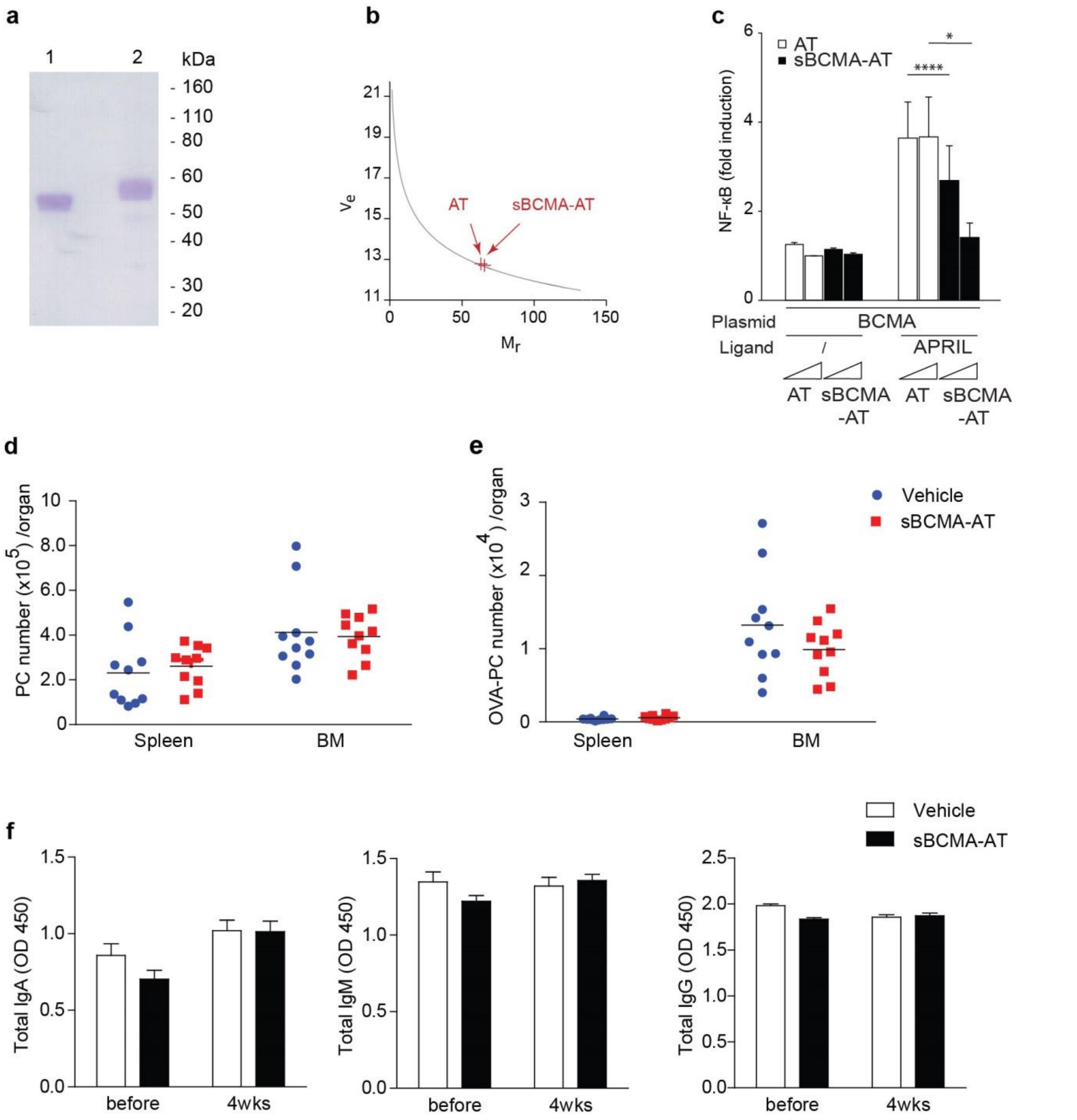

Supplementary Figure 6. Features of recombinant sBCMA-AT. (**a**) Purified AT (lane 1) and sBCMA-AT (lane 2) were separated by SDS-PAGE and stained with Coomassie. (**b**) AT and sBCMA-AT were analyzed by gel-filtration. (**c**) HEK293T cells were transfected with human BCMA and activated with mouse APRIL as indicated. sBCMA-AT (100 and 500 ng ml^{-1}) or AT (100 and 500 ng ml^{-1}) as a control were was applied. Combined data of 5 independent experiments (mean±s.e.m, paired t-test). (**d-f**) 3 months old NZB/W mice were immunized intraperitoneally (OVA on alum). At 4 months, mice were treated for 4 weeks intraperitoneally with recombinant mouse sBCMA-AT resulting in a serum concentration of sBCMA-AT of 82 µg ml^{-1} (±16 µg ml^{-1}). Total number of plasma cells (**d**), and of OVA-specific plasma cells (**e**) were determined in the spleen and bone marrow (BM). Immunoglobulin levels were measured by ELISA, mean±s.e.m. (**f**).

Supplementary Figure 7

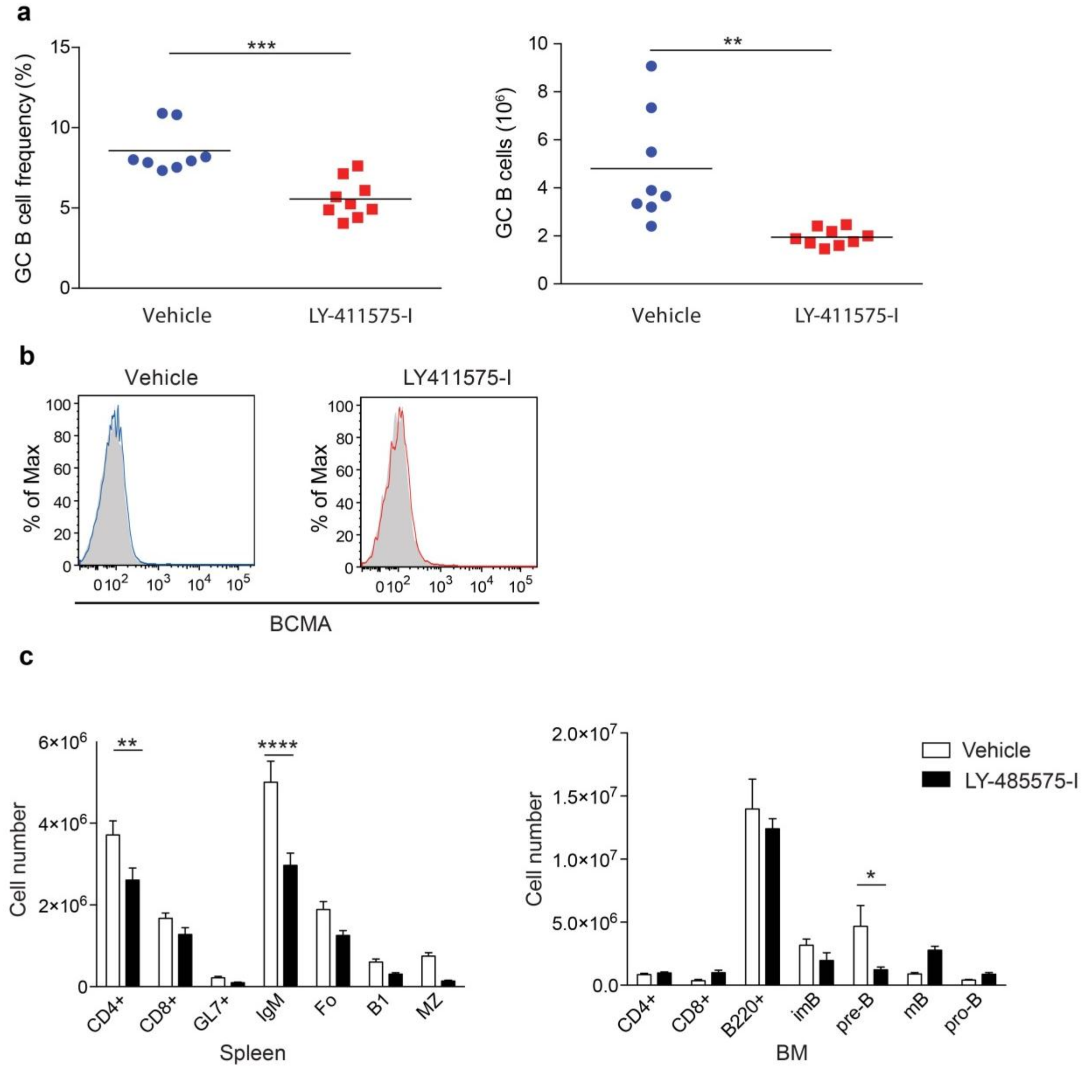

Supplementary Figure 7. Effects of γ-secretase inhibition in vivo on different immune cell subsets. (a,b) Immunized (OVA-LPS in alum) C57/BL6 mice were treated with the γ-secretase inhibitor LY-411575-I or vehicle. Splenocytes were harvested and germinal center B cells (Fas^{high} CD38^{int}) were identified by FACS. (**a**) Frequency of GC cells (left panel) in regard to the whole B cell population in treated and untreated mice. (unpaired t test, p=0.0003) and absolute number of GC B cells (right panel) in treated and untreated mice (unpaired t test, p=0.0023). (**b**) BCMA expression on GC B cells in treated and untreated mice. (**c**) NZB/W mice were treated with LY-411575-I for 7 days. Absolute number of T cells and B-cell subsets in the spleen and in the bone marrow (BM). (n=10) mean±s.e.m., 2way Anova, Bonferonni’s multiple comparison test).

Supplementary Figure 8

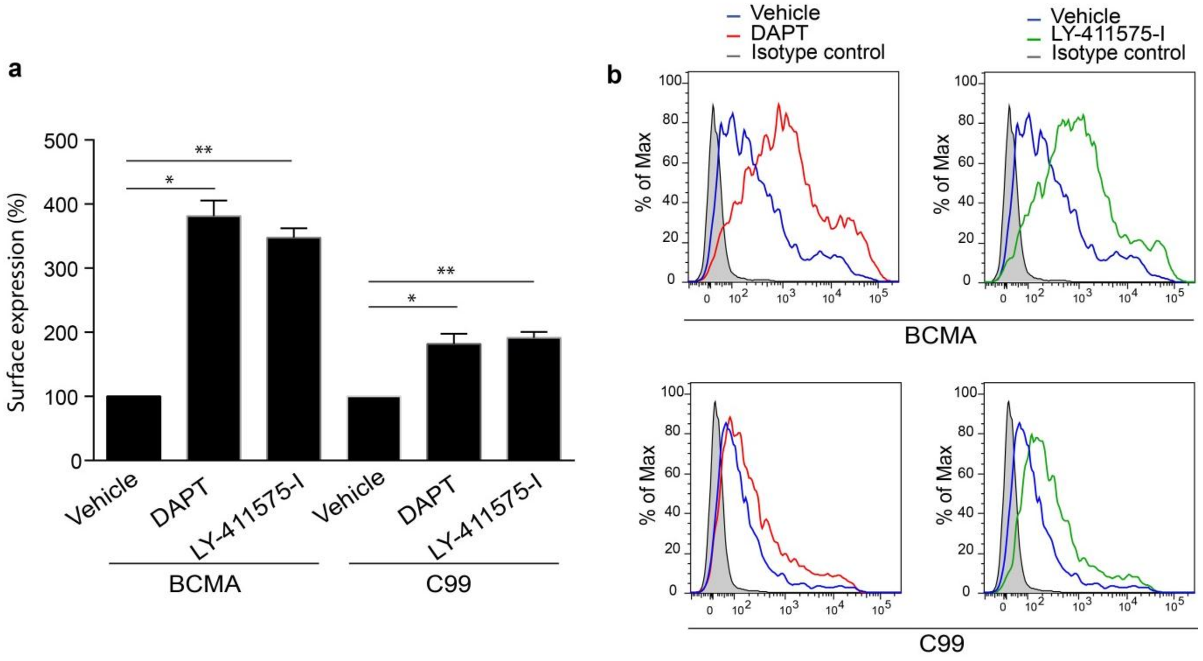

Supplementary Figure 8. LY-411575-I inhibits shedding of BCMA and C99. HEK293T cells were transfected with human BCMA or with the established γ-secretase substrate C99 and treated overnight with either DAPT (1 µM), with LY-411575-I (100 nM) or with the appropriate vehicle. Surface expression of BCMA or C99 was measured in by flow cytometry. (**a**) Surface expression of human BCMA or C99 upon DAPT and LY-411575-I treatment. Mean±s.e.m. of 6 replicates, *, p <0,05; **, p<0,01, unpaired t-test. (**b**) Representative histograms of BCMA and C99 surface expression upon DAPT (1 µM) and LY-411575-I (100 nM) treatment.

Supplementary Figure 9

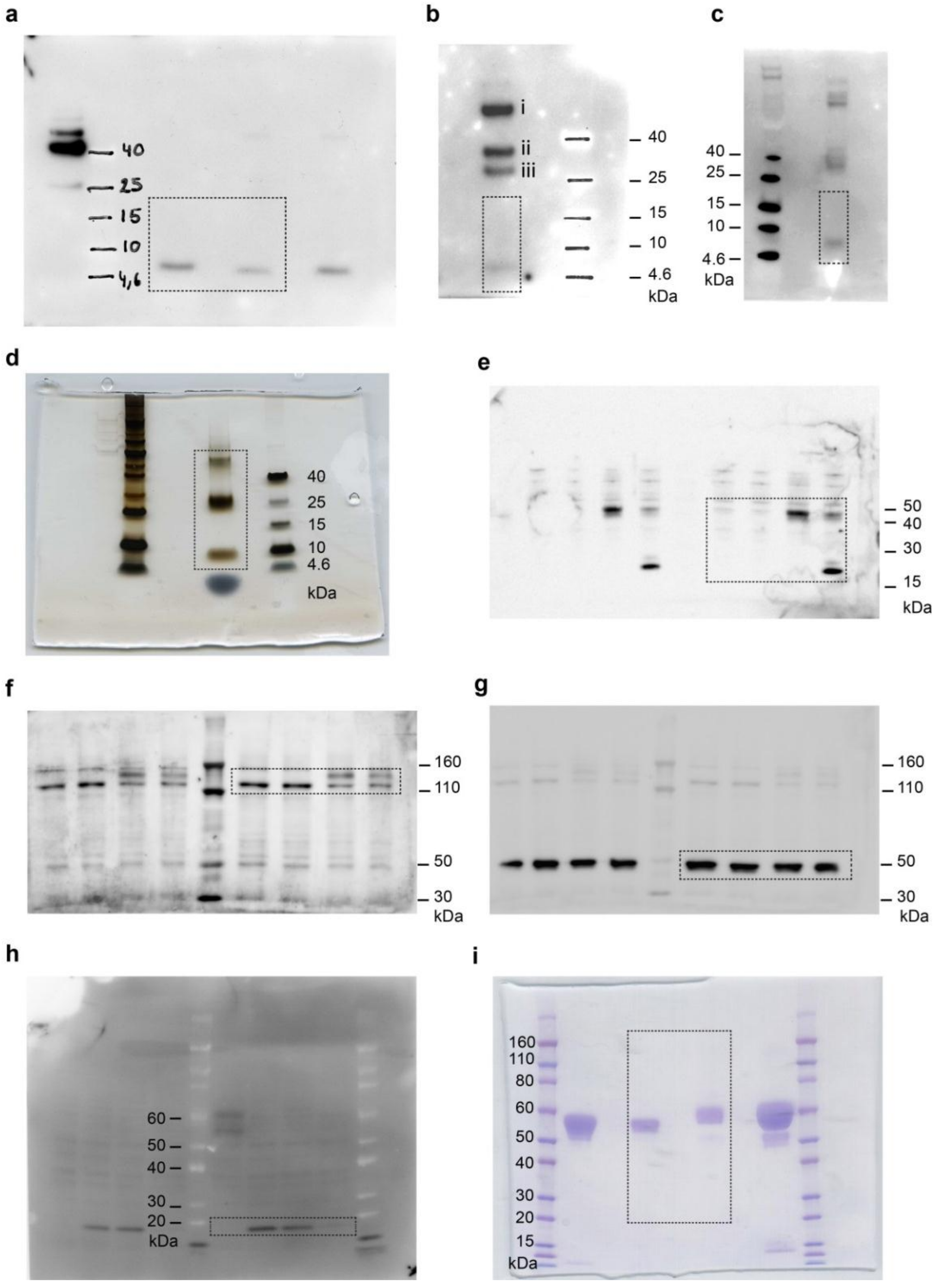

Supplementary Figure 9. Original images of the cropped blots and gels shown in the paper. BCMA Western blot from supernatant of plasmacytoma cells (**a**), serum (**b**), and from supernatant of human purified B cells (**c**) of Fig. 1f. The bands indicated in b contained : IgG heavy chain (i), complement component1 subA (ii) and IgG light chain (iii) as seen by mass-spectrometry. (**d**) Silver gel of immunoprecipated sBCMA from supernatant of plasmocytoma cells, Fig. 1g. (**e**) PSEN1 Western blot of Fig. 4c. (**f**) Nicastrin Western blot of Fig. 4c (**g**) β-actin Western blot of Fig. 4c. (**h**) BCMA Western blot of Fig. 4c. (**i**) Coomassie gel of supplementary Fig. 6.a.

Supplementary Table 1. Clinical data of patients included in this study

Age (year)	sex	Diagnosis	Treatment	Related Figure
52	F	RRMS	GA + GCS	Supplementary Figure 1c
41	F	RRMS	GA + GCS	Supplementary Figure 1c
50	M	RRMS	GCS	Supplementary Figure 1c
40	M	RRMS	GCS	Supplementary Figure 1c
39	M	RRMS	AZA + GCS	Supplementary Figure 1c
29	F	RRMS	GCS	Supplementary Figure 1c
51	F	RRMS	GCS	Supplementary Figure 1c
29	M	RRMS	IFN-beta 1a + GCS	Supplementary Figure 1c
32	F	CIS	GCS	Supplementary Figure 1c
33	F	RRMS	GA + GCS	Supplementary Figure 1c
50	F	RRMS	NTZ	Supplementary Figure 1b
33	F	RRMS	NTZ	Supplementary Figure 1b
34	M	RRMS	NTZ	Supplementary Figure 1b
28	M	RRMS	NTZ	Supplementary Figure 1b
50	F	RRMS	NTZ	Supplementary Figure 1b
56	M	RRMS	NTZ	Supplementary Figure 1b
37	F	RRMS	NTZ	Supplementary Figure 1b
38	F	RRMS	NTZ	Supplementary Figure 1b
31	F	RRMS	NTZ	Supplementary Figure 1b
56	M	RRMS	NTZ	Supplementary Figure 1b
24	F	RRMS	no treatment	Figure 1a,b,c
26	F	CIS	no treatment	Figure 1a,b,c
28	M	RRMS	no treatment	Figure 1a,b,c
34	F	RRMS	no treatment	Figure 1a,b,c
24	F	CIS	no treatment	Figure 1a,b,c
43	F	CIS	no treatment	Figure 1a,b,c
33	F	RRMS	no treatment	Figure 1a,b,c
38	F	RRMS	no treatment	Figure 1a,b,c
32	F	RRMS	no treatment	Figure 1a,b,c
44	F	RRMS	no treatment	Figure 1a,b,c
37	F	CIS	no treatment	Figure 1a,b,c
45	F	RRMS	no treatment	Figure 1a,b,c
62	F	RRMS	no treatment	Figure 1a,b,c
51	M	RRMS	no treatment	Figure 1a,b,c
21	F	RRMS	no treatment	Figure 1a,b,c
59	F	RRMS	no treatment	Figure 1a,b,c
42	F	RRMS	no treatment	Figure 1a,b,c
40	F	CIS	no treatment	Figure 1a,b,c
21	M	CIS	no treatment	Figure 1a,b,c
33	F	CIS	no treatment	Figure 1a,b,c

53	M	RRMS	no treatment	Figure 1a,b,c
32	M	RRMS	no treatment	Figure 1a,b,c
24	F	CIS	no treatment	Figure 1a,b,c
37	F	RRMS	no treatment	Figure 1a,b,c
38	F	RRMS	no treatment	Figure 1a,b,c
45	F	RRMS	no treatment	Figure 1a,b,c
24	F	RRMS	no treatment	Figure 1a,b,c
27	F	RRMS	no treatment	Figure 1a,b,c
22	F	RRMS	no treatment	Figure 1a,b,c
26	F	RRMS	no treatment	Figure 1a,b,c
62	F	SPMS	no treatment	Figure 1a,b,c
46	F	SPMS	no treatment	Figure 1a,b,c
59	M	SPMS	no treatment	Figure 1a,b,c
62	M	SPMS	no treatment	Figure 1a,b,c
39	M	SPMS	no treatment	Figure 1a,b,c
45	F	SPMS	no treatment	Figure 1a,b,c
54	F	SPMS	no treatment	Figure 1a,b,c
46	F	Neuroborreliosis	no treatment	Figure 1b,c
69	F	Neuroborreliosis	no treatment	Figure 1b,c
66	M	Neuroborreliosis	no treatment	Figure 1b,c
55	M	Neuroborreliosis	no treatment	Figure 1b,c
61	M	Neuroborreliosis	no treatment	Figure 1b,c
45	F	SLE	no treatment	Figure 1d,e,f
47	F	SLE	no treatment	Figure 1d,e,f
33	F	SLE	no treatment	Figure 1d,e,f
27	F	SLE	no treatment	Figure 1d,e,f
62	F	SLE	no treatment	Figure 1d,e,f
38	F	SLE	no treatment	Figure 1d,e,f
40	F	SLE	no treatment	Figure 1d,e,f
40	F	SLE	no treatment	Figure 1d,e,f
31	F	SLE	no treatment	Figure 1d,e,f
39	F	SLE	no treatment	Figure 1d,e,f
26	F	SLE	no treatment	Figure 1d,e,f
22	F	SLE	no treatment	Figure 1d,e,f
20	F	SLE	no treatment	Figure 1d,e,f
22	F	SLE	no treatment	Figure 1d,e,f
43	F	SLE	no treatment	Figure 1d,e,f
55	F	SLE	no treatment	Figure 1d,e,f
21	F	SLE	no treatment	Figure 1d,e,f
29	F	SLE	Pred, HCQ	Figure 1d,e,f
38	F	SLE	Pred, HCQ, AZA	Figure 1d,e,f

39	F	SLE	Pred, HCQ, CYC	Figure 1d,e,f
54	F	SLE	Pred, AZA	Figure 1d,e,f
47	F	SLE	Pred, HCQ	Figure 1d,e,f
63	F	SLE	HCQ, AZA	Figure 1d,e,f
28	F	SLE	HCQ	Figure 1d,e,f
38	F	SLE	Pred	Figure 1d,e,f
36	F	SLE	Pred, MMF	Figure 1d,e,f
41	F	SLE	Pred, HCQ	Figure 1d,e,f
30	F	SLE	AZA	Figure 1d,e,f
60	F	SLE	HCQ, AZA	Figure 1d,e,f
25	F	SLE	HCQ	Figure 1d,e,f
31	F	SLE	Pred, HCQ, MMF	Figure 1d,e,f
27	F	SLE	Pred, CYC	Figure 1d,e,f
45	F	SLE	Pred, HCQ, CYC	Figure 1d,e,f
22	F	SLE	Pred, HCQ, CYC	Figure 1d,e,f
28	F	SLE	Pred, HCQ, AZA	Figure 1d,e,f
23	F	SLE	Pred	Figure 1d,e,f
25	F	SLE	Pred, CYC	Figure 1d,e,f
31	F	SLE	Pred, AZA	Figure 1d,e,f
32	F	SLE	Pred	Figure 1d,e,f
38	F	OND (Sensineuronal hearing deficit)		Figure 1a,b
68	F	OND (Cerebrovascular disease)		Figure 1a,b
29	M	OND (Sensory symptoms)		Figure 1a,b
34	F	OND (Sensory symptoms)		Figure 1a,b
45	F	OND (Migraine)		Figure 1a,b
26	M	OND (Sensory symptoms)		Figure 1a,b
41	F	OND (Sensory symptoms)		Figure 1a,b
19	F	OND (Vertigo)		Figure 1a,b
31	M	OND (Sensory symptoms)		Figure 1a,b
35	F	OND (Syringomyelia)		Figure 1a,b
51	M	OND (Spinal stenosis)		Figure 1a,b
30	F	OND (Neurastenia)		Figure 1a,b
65	M	OND (Alcohol-related spastic paraparesis)		Figure 1a,b
37	F	OND (Sensory symptoms)		Figure 1a,b
49	F	OND (Hearing deficit)		Figure 1a,b
53	F	OND (Depression)		Figure 1a,b
52	F	OND		Figure 1a,b

33	F	Fatigue		Figure 1b,c
40	M	Bipolar disorder		Figure 1b,c
32	M	Schizophrenia		Figure 1b,c

Abbreviations used in this table: GA: Glatiramer Acetate; GCS: Glucocorticosteroid, NTZ: Natalizumab, PRED: Prednisolon, HCQ: Hydroxychloroquine, AZA: Azathioprine, MMF: Mycophenolate mofetil, CYC: Cyclophosphamide.

Supplementary Table 2. Antibodies used in the study

Antibody	Source	Dilution
anti-hBCMA (C12A3.2)	Biogen Idec	FC: 1µg ml^{-1}<br>WB: 1µg ml^{-1}
anti-hBCMA (A7D12.2)	Biogen Idec	FC: 5µg ml^{-1}
anti-presenilin (PSEN1)	Epitomics	WB: 1:2500
anti-nicastrin (N1660)	Sigma-Aldrich	WB: 1:5000
anti-CD40L, FITC (TRAP1)	BD PharMingen	FC: 1:40
anti-CD138, FITC (B-A38)	Diaclone	FC: 1:10
anti-CD19, PE (HIB19)	BD PharMingen	FC: 1/40
anti-CD19, Cy7 (HIB19)	Ebioscience	FC: 1:40
anti-CD27, Cy7 (O323)	Ebioscience	FC: 1:20
anti-CD38, eFluor® 450 (HB7)	Ebioscience	FC: 1:20
anti-CD138, PE (281-2)	BD Biosciences	FC: 1:100
anti-kappa, Pacific Orange (187.1)	DRFZ	FC: 1:50
anti-CD21 (7E9)	BioLegend	FC: 1:100
anti-CD23 (B3B4)	BioLegend	FC: 1:100
anti-CD24 (M1/69)	BD Biosciences	FC: 1:200
anti-CD93 (AA4.1)	BioLegend	FC: 1:50
anti-CD117 (2B8)	BD Biosciences	FC: 1:200
anti-IgM (RMM-1)	BioLegend	FC: 1:20
anti-B220 (RA3-6B2)	DRFZ	FC: 1:400
anti-IgD (11-26c)	DRFZ	FC: 1:200
anti-GL-7 (GL-7)	DRFZ	FC: 1:200
anti-CD4 (GK1.5)	DRFZ	FC: 1:200
anti-CD8 (53-6.7)	BioLegend	FC: 1:50
anti-FLAG™ (M2)	Sigma-Aldrich	FC: 5µg ml^{-1}<br>ELISA: 5µg ml^{-1}
goat-anti-mouse IgG2b, Alexa Fluor® 647	Invitrogen	FC: 1:500
goat-anti-mouse Ig, PE	Dako	FC: 1:50
goat-anti-mouse IgG, HRP (True blot®)	Ebioscience	WB: 1:1000
anti-rabbit IgG, HRP	Promega	WB: 1:2500-5000
mouse anti-C99 (4G8)	Covance	FC: 2 µg ml^{-1}

Abbreviations used in this table: WB: Western blot; FC: flow cytometry; DRFZ: Deutsches Rheuma-Forschungszentrum
